# Identification and characterization of key residues in Zika virus envelope protein for virus assembly and entry

**DOI:** 10.1080/22221751.2022.2082888

**Published:** 2022-06-10

**Authors:** Xiao Ma, Zhenghong Yuan, Zhigang Yi

**Affiliations:** a Key Laboratory of Medical Molecular Virology (MOE/NHC/CAMS), School of Basic Medical Sciences, and Shanghai Institute of Infectious Disease and Biosecurity, Fudan University, Shanghai, People’s Republic of China; b Shanghai Public Health Clinical Center, Fudan University, Shanghai, People’s Republic of China

**Keywords:** Zika virus (ZIKV), flavivirus, envelope protein, glycoprotein, viral egress, viral entry, transcomplemented particle system, mutagenesis

## Abstract

Zika virus (ZIKV), a family member in the *Flavivirus* genus, has re-emerged as a global public health concern. The envelope (E) proteins of flaviviruses play a dual role in viral assembly and entry. To identify the key residues of E in virus entry, we generated a ZIKV *trans*-complemented particle (ZIKV_TCP_) system, in which a subgenomic reporter replicon was packaged by *trans*-complementation with expression of CprME. We performed mutagenesis studies of the loop regions that protrude from the surface of the virion in the E ectodomains (DI, DII, DIII). Most mutated ZIKV_TCPs_ exhibited deficient egress. Mutations in DII and in the hinge region of DI and DIII affected prM expression. With a bioorthogonal system, photocrosslinking experiments identified crosslinked intracellular E trimers and demonstrated that egress-deficient mutants in DIII impaired E trimerization. Of these mutants, an E-trimerization-dead mutation D389A that nears the E-E interface between two neighbouring spikes in the immature virion completely abolished viral egress. Several mutations abolished ZIKV_TCPs_’ entry, without severely affecting viral egress. Further virus binding experiments demonstrated a deficiency of the mutated ZIKV_TCPs_ in virus attachment. Strikingly, synthesized peptide containing residues of two mutants (268-273aa in DII) could bind to host cells and significantly compete for viral attachment and interfere with viral infection, suggesting an important role of these resides in virus entry. Our findings uncovered the requirement for DIII mediated-E trimerization in viral egress, and discovered a key residue group in DII that participates in virus entry.

## Introduction

Zika virus (ZIKV), a re-emerging human pathogen, belongs to the *Flavivirus* genus, which also include members such as dengue virus (DENV), yellow fever virus (YFV) and West Nile virus (WNV) [1]. ZIKV was first isolated in Uganda in 1947 and re-emerged recently in Asia and America [2,3]. ZIKV infection causes mild flu-like symptoms to serious birth defects and neurological diseases such as human microcephaly, congenital malformations and Guillain-Barré syndrome [4]. There is currently no specific vaccine or treatment for ZIKV infection.

ZIKV is a single-stranded, positive-sense RNA virus. Its genome is translated into a polypeptide that is processed by virus and host proteases to produce structural proteins (C, prM and E) and non-structural proteins (NS1, NS2A, NS2B, NS3, NS4A, NS4B, and NS5) [5]. The structural proteins of ZIKV assemble the viral particle and the nonstructural proteins are used for virus replication, virion assembly and evasion of host immune response [6,7]. The capsid (C) protein, prM and E engage viral genomic to form immature viral particles on the surface of endoplasmic reticulum (ER) [8]. In the immature virus particles, the prM protein forms a heterodimer with the E protein [9,10]. Trimeric prM-E heterodimers assemble into spikes in the immature virion and each immature virus particle contains 60 spikes [9,11,12]. The immature virus particle is further transported through the exocytotic pathway. In the acidic trans-Golgi network (TGN), a transition from trimeric prM-E heterodimer to E homodimer occurs, exposing the pr/M cleavage site. Cleavage of prM by furin protease results in maturation of the virion. The mature virus particle is composed of 90 E protein homodimers that are parallel to the viral membrane [11–13].

ZIKV E protein is the main membrane protein on the virus surface. It is composed of an N-terminal ectodomain and the C-terminal transmembrane helix (TM). The ectodomain includes the central domain (DI), a dimerization domain (DII), and an immunoglobulin-like domain (DIII). Flaviviruses invade cells through E-mediated receptor binding [13,14]. Although several host factors such as DC-SIGN, AXL and TYRO3 have been shown to mediate viral entry, the molecular mechanisms of flavivirus entry are also poorly understood [13,15–21].

Many residues of E have been reported to affect entry [22]. Glycosylation in the envelope (E) protein (N154) contribute to periphery viral infection in an animal model, probably through interacting with DC-SIGN [18]. Peptides containing N154 bind to fibroblasts and primary neuronal cells [23]. Polyubiquitination of E K38 and K281 mediates interaction with TIM1 for virus attachment [24]. Antibodies against all three domains can neutralize viral infection [25]. Loop regions in DIII have been proposed to contribute to virus binding to receptors [26–31]. The antibodies against DIII [32] or residues outside DIII [33,34] may interfere with viral attachment via different mechanisms [35]. Flag tag insertion and anti-Flag antibody-mediated blocking experiments also have shown an important role of residues in DI and DII in virus entry [36]. Thus far, the key E residues that participate in receptor binding remain elusive. Identification of the key E residues and characterizing the roles of these resides in virus attachment and entry would help to decipher the molecular mechanisms of virus entry.

Given the dual requirements for E protein in virion assembly and virus entry, it is difficult to dissect the residues of E solely involved in virus entry. To identify the key resides that participate in virus entry, we first generated a ZIKV *trans*-complemented particle (ZIKV_TCP_) system, in which a ZIKV subgenomic replicon RNA bearing a secreted Gaussian luciferase (sGluc) reporter was packaged by CprM-E complementation. By using this ZIKV_TCP_ system, we performed mutagenesis of the E loop regions that protrude from the surface of the virion and may serve as putative receptor-binding sites. We found that most mutations caused defects in viral egress, including mutations in DII and in the hinge region of DI and DIII that affected prM expression and mutations in DIII that affected E trimerization. We found mutations that impaired virus attachment without severely affecting virus egress. Further virus binding and peptide competing experiments demonstrate that residues 268–273 in DII contribute to virus attachment.

## Material and methods

### Plasmids

To generate the plasmids phCMV-CprM-E, phCMV-prM-E, the coding regions of CprM-E and prM-E were amplified from a ZIKV infectious clone [37] and then inserted into the XhoI/NotI site in the phCMV plasmid, respectively. HA tag (YPYDVPDYA) was introduced after the residue 147 in E as previously reported [36] by fusion PCR-mediated mutagenesis to get the phCMV-CprM-E^HA^ and phCMV-prM-E^HA^, respectively. Three-Alanine (AAA) mutations were introduced into E in the plasmid phCMV-CprM-E^HA^ by fusion PCR-mediated mutagenesis to get the plasmids phCMV-CprM-E^HA^-Mutants. The suppressor tRNA plasmid (pSVB.Yam) and the amino-acyl tRNA synthetase plasmid for p-azido-L-phenylalanine (pcDNA.RS) were kindly gifted by Thomas P. Sakmar (Rockefeller University) and have been described previously [38]. The TAG was introduced into E in the plasmid phCMV-CprM-E^HA^ by fusion PCR-mediated mutagenesis. The L107TAG mutation was introduced into plasmids phCMV-CprM-E^HA^-mutants (Mut8 to Mut43) to get the plasmids phCMV-CprM-E^HA^-L107TAG-mutants (Mut8 to Mut43), respectively. The infectious clones of ZIKV C7 and C7-Gluc plasmid has been described previously [37]. The coding sequences of the HA tag (YPYDVPDYA) and the Flag tag (DYKDDDDK) were inserted after the residue 147 in E in the C7 infectious clone by fusion PCR-mediated mutagenesis to get the plasmids C7.E^HA^ and C7.E^Flag^, respectively. The details of the mutation were described in Supplementary Table 1.

### Virus and ZIKV_TCP_

ZIKV C7, C7-Gluc were generated by electroporation of *in vitro*-transcribed viral RNAs into Vero cells. The C7.E^HA^ and C7.E^Flag^ were generated by transfection of *in vitro*-transcribed viral RNAs into Vero cells with transfection reagent (TransIT®-mRNA Transfection Kit, Mirus, MIR 2250). The virus titres were determined in Vero cells by plaque assay as described [37].

To generate ZIKV_TCPs_, sgZIKV-sGluc-Vero cells (0.75 × 10^5^) in 48-well plate were transfected with 0.26 μg phCMV-CprM-E^HA^ (WT), phCMV-prM-E^HA^ (ΔC) or phCMV-CprM-E^HA^-mutant plasmids by TransIT®-LT1 Transfection Reagent (Mirus, MIR 2306). Four days later, the supernatant was collected and centrifuged at 500 g for 10 min to remove the debris and stored at −20°C.

### ZIKV_TCP_ concentration

About 3.6 × 10^6^ sgZIKV-sGluc-Vero cells in 10-cm dish were transfected with 15 μg phCMV-CprM-E^HA^ or the phCMV-CprM-E^HA^ mutants plasmids using the TransIT®-LT1 Transfection Reagent (Mirus, MIR 2306), 4 days later, the supernatants were collected into a 15 mL centrifuge tube and centrifuge at 1000× *g* for 10 min to remove debris. About 11.2 ml clarified supernatants were loaded on the top of 2 mL 20% sucrose cushion (W/V in PBS). Centrifugation was performed with SW41 rotor (BECKMAN COULTER, 331372) at 247,600× *g* for 4 h at 4°C with no brake. After gently removal of the supernatants, ZIKV_TCP_ pellet was resuspended in 300 μl PBS, aliquot, and stored at −80°C.

### Virus binding assay

Vero cells were infected with C7.E^HA^ (MOI, 6) or the C7.E^HA^-normalized ZIKV_TCPs_ at 4°C for 2 h. After being washed six times with PBS, the cells were either harvested for quantification of viral RNA or fixed for immunostaining.

For peptide competing for viral infection, peptides P1 (VLGSQ EGAVH TALAG **ALEAEM** DGAKG RLFSG HLKCR LKMDK LRL) and P1-Mut (VLGSQ EGAVH TALAG **AAAAAA** DGAKG RLFSG HLKCR LKMDK LRL) were synthesized by GL Biochem (Shanghai) Ltd and dissolved in PBS to make a 0.5 mg/ml stock. Vero cells (0.75 × 10^5^) were seeded onto 48wps and incubated at 37°C overnight. The cells were washed with PBS and incubated with 10 μM P1 or P1-Mut for 2h at 4°C, then infected with C7-Gluc (MOI, 0.01) or C7.E^HA^-normalized ZIKV_TCP_ for 2h at 4°C. The supernatants were discarded, and the cells were added with fresh media. The luciferase activity was measured 4 days later.

For peptide competing for virus binding, Vero cells are digested by trypsin, resuspended in PBS with 1% FBS and adjusted to a cell concentration of 1.2 × 10^6^ cells/ml. About 1 ml of cell suspension was incubated with various concentrations of P1 and P1-Mut at 4°C for 2 h, and then infected with C7-Gluc (MOI, 0.01) or C7 (MOI, 0.01) for 2h at 4°C. After been washed with PBS for six times by centrifugation at 1100*g* for 5 min, RNAs were extracted by TRIzol reagent and subjected to reverse transcription and Real-time PCR assay.

### Peptide binding assays

Peptides FITC-P1 (FITC-VLGSQ EGAVH TALAG **ALEAEM** DGAKG RLFSG HLKCR LKMDK LRL), FITC-P1-Mut: (FITC-VLGSQ EGAVH TALAG **AAAAAA** DGAKG RLFSG HLKCR LKMDK LRL), FITC-P2: (FITC-GRLIT ANPVI TESTE NS**KMM** LELDP PFG) and FITC-P2-Mut (FITC-GRLIT ANPVI TESTE NS**AAA** LELDP PFG) were synthesized by GL Biochem (Shanghai) Ltd and dissolved in PBS to make a 0.5 mg/ml stock. Various concentrations of peptides were incubated with Vero cells for 2h at 4°C. Then, the peptides were removed, and the cells were washed with PBS four times and then fixed with 4% paraformaldehyde for 15 min and observed by fluorescence microscopy.

For flow cytometry assay, Vero cells are digested by trypsin, resuspended in PBS and adjusted to a cell concentration of 1.2 × 10^6^ cells/ml. About 1 ml of cell suspension was incubated with various concentrations of FITC-P1, FITC-P1-Mut, FITC-P2 and FITC-P2-Mut at 4°C for 2 h. The cells were washed with PBS three times by centrifugation at 1100*g* for 5 min and then fixed in 400 μl 3.5% paraformaldehyde for 5 min. After been further washed with PBS for three times, samples were analyzed by Attune NxT Acoustic Focusing Cytometer.

### Fluorescent antibody staining

Fixed cells were blocked by incubating with 3% BSA in PBS for 2 h at room temperature. Then, the cells were incubated with anti-HA (1:300) and anti-CD44 (1:300) antibodies at 4°C overnight. After being washed three times with PBS containing 3%BSA, samples were incubated with Alexa 488- and Cy3-conjugated secondary antibody at 37°C for 1 h. After being washed thrice with PBS, samples were incubated with 2 ml PBS containing 2 μl Hoechst 33342 (Invitrogen™, H3570) for 3 min to stain the nucleus. After being mounted on the coverslips, the cells were observed using a confocal laser scanning microscope (TCS-NT; Leica, Heidelberg, Germany). Images were acquired and analyzed by using Image J software as previously described [39].

### *In-vivo* photo crosslinking

Photo crosslinking was carried out as previously described [38]. Briefly, 7.5 × 10^5^ Vero cells on 6-cm dish were cotransfected with 4.3 μg CprM-E expressing plasmids, 4.3 μg pSVB.Yam and 1.2 μg pcDNA.RS by TransIT-LT1 (Mirus) at a ratio of 1:3 (plasmid: reagent). Nine hours after transfection, the medium was changed to DMEM containing 10% FBS and 0.5 mM azF. Forty-eight hours post transfection. The cells were washed with PBS and then irradiated on ice under 365 nm UV light by a UV lamp (WFH-204B, Shanghai Jingke) in a dark room for 30 min. The UV light energy given out by the lamp is 3.405 Ev and the sample is 20 mm away from the UV lamp. Then, the cells were lysed with 300 µl IP buffer (50 mM TrisCl [pH 7.5], 1 mM EDTA, 15 mM MgCl_2_, 10 mM KCl, 1% Triton X-100, proteinase inhibitor [Roche]). After disruption by passing through a 27-gauge needle 20 times and clarification by centrifugation at 15,000*g* for 10 min at 4°C, 300 μl of the soluble fraction was incubated overnight at 4°C with 10 µl anti-HA magnetic beads (Pierce™, 88837). Beads were washed 4 times in 500 µl wash buffer (50 mM TrisCl [pH 7.5], 1 mM EDTA, 15 mM MgCl_2_, 10 mM KCl, 1% Triton X-100), and then lysed in 2 × SDS loading buffer. The samples were boiled for 10 min and then analyzed by Western blotting.

### Statistical analysis

Statistical analysis was performed with the GraphPad Prism 8 software. Specific tests are described in the figure legends.

## Results

### Generation of ZIKV *trans*-complemented particles (ZIKV_TCPs_)

**Figure 1.**
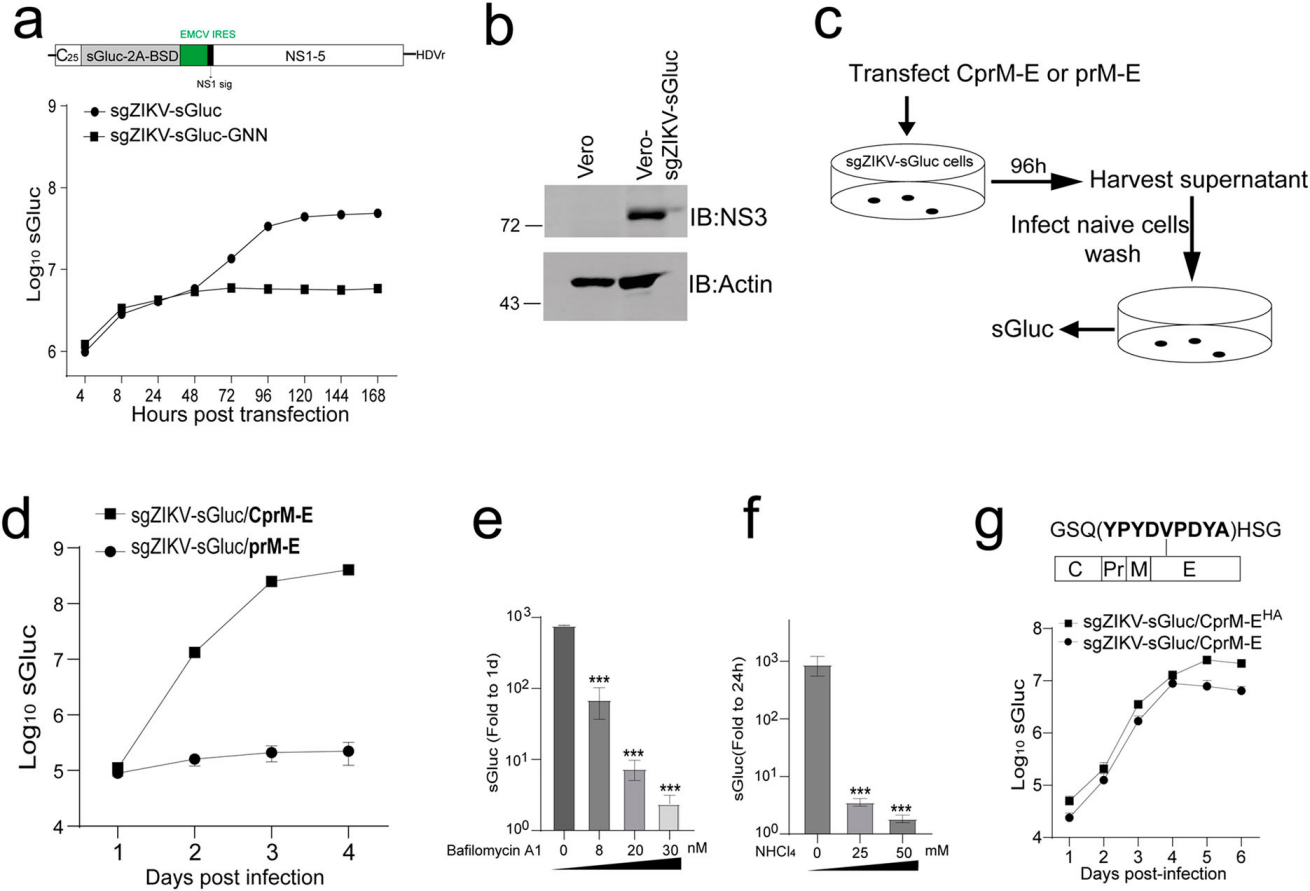
Generation and characterization of ZIKV *trans*-complemented particles (ZIKV_TCPs_). (a) Replication of ZIKV subgenomic replicon. Upper panel, schematic of ZIKV subgenomic. C_25_, coding sequence of the first 25-amino acids of capsid. sGluc, secreted *Gaussia* luciferase. 2A, FMDV 2A peptide. BSD, blasticidin resistance gene. NS1.sig, signal peptide of NS1. HDVr, HDV ribozyme. Bottom panel, replication of sgZIKV-sGluc and sgZIKV-sGluc-GNN with NS5 lethal mutation. The *in vitro*-transcribed RNAs were transfected into Vero cells. Supernatants were harvested at various time points and the luciferase activities were determined. Mean values ± SDs are shown (*n* = 3). (b) Generation of the sgZIKV-sGluc stable cell line. SgZIKV-sGluc RNAs were transfected into Vero cells and grown in medium with 5 μg/ml basticidin, and after 15 passages, the cell lysates were analyzed by western blotting with the indicated antibodies. The values to the left of the blots are molecular sizes in kilodaltons. (c and d) Generation of ZIKV_TCP_. (c) Schematic of the experiment design. Plasmids expressing ZIKV CprM-E or prM-E were transfected into Vero-sgZIKV-sGluc cells. After 96 h, the conditioned medium was harvested and used to infect naïve Vero cells. (d) About 7.5 × 10^4^ Vero cells in the 48-well plate format were infected with 80 μl condition medium for 1 day. Then cells were washed thrice with PBS and fresh media was added. The supernatants were collected at various time points post infection and the luciferase activity in the supernatants was measured. Mean values ± SDs are shown (*n* = 3). (e and f) Inhibition of ZIKV_TCP_ infection by Bafilomycin and NHCl_4._ Vero cells were infected with ZIKV_TCP_ in the presence of Bafilomycin (e) and NHCl_4_ (f) at various concentrations as described in d. At 1-day post infection, cells were washed with PBS and fresh media was added. Supernatants were collected at 1-day and 4-day post infection and luciferase activity were determined. The relative luciferase activity at 4-day post infection relative to 1-day post infection was plotted. Mean values ± SDs are shown (n = 3). Statistical analysis was performed between the treated groups and the control-treated (0) group (***P < 0.001; two-tailed, unpaired *t*-test). (g) Infection of ZIKV_TCP_^HA^. ZIKV_TCP_^HA^ with HA-tagged E protein (upper panel) was generated as described in (c) by transfecting plasmid expressing CprM-E^HA^. ZIKV_TCPs_ were used to infect naïve Vero cells as described in (c). The supernatants were harvested at the indicated time points and luciferase activates were measured and plotted. Mean values ± SDs are shown (*n* = 3).

To dissect the roles of E residues in virus egress and entry, we attempted to generate a *trans*-complemented particle system, in which the viral structural proteins CprM-E are expressed in ZIKV subgenomic replicon cells and the viral subgenomic replicon RNA is then packaged by the CprM-E *trans*-complementation. *Trans*-complemented particle system is a widely used strategy to dissect the assembly and entry process in flavivirus, especially when some E mutations abolished virus rescue in the reverse genetic systems and thus it is impossible to generate an E-mutated virus [40–44]. *Trans*-complemented particle system is a widely used molecular biology tool to dissect the assembly and entry process in flavivirus, although it doesn’t quite represent a real virus [42,45,46]. First, we devised a subgenomic replicon based on an infectious clone of ZIKV MR766 [37] with a similar strategy as reported before [47] (Figure 1(a)). The coding region of the structural proteins was removed and replaced with a cassette expressing a secreted *Gaussia* luciferase (sGluc), FMDV 2A peptide (2A) and blasticidin resistant gene (BSD). The coding sequence of the first 25 residues of capsid was retained. The EMCV IRES was inserted before the open reading frame of the polypeptide of viral non-structural proteins NS1-NS5. The C-terminal 24 resides of E was retained to function as the signal peptide (NS1 sig) of NS1. The self-cleaving RNA sequence (HDV ribozyme, HDVr) was used to generate the genuine 3’-terminal nucleotide of the viral genome after *in vitro* transcription. To assess the replication of the subgenomic RNA, the *in-vitro*-transcribed replicon RNA (sgZIKV-sGluc) and a mutant bearing the RNA-dependent-RNA polymerase inactive mutations (sgZIKV-sGluc-GNN) were transfected into Vero cells. Luciferase activities in the supernatants were measured at various time points post transfection. Comparing with sgZIKV-sGluc-GNN RNA, the sgZIKV-sGluc RNA exhibited robust replication, whereas the sgZIKV-sGluc-GNN only supported initial translation of viral RNA (Figure 1(a)). Then we attempted to generate a stable replicon cell line. Vero cells were transfected with sgZIKV-sGluc RNA and then grown in medium with 5 μg/ml basticidin. After 15 passages, the surviving cells were pooled and the expression of viral NS3 was verified by Western blot analysis (Figure 1(b)).

Next, we tried to use the structural proteins CprM-E provided *in trans* to package the replicon RNA. We transfected plasmids expressing CprM-E into the Vero-sgZIKV-sGluc cells. The CprM-E would be processed by host signalase and the viral serine protease into C, prM and E [5]. We also took prM-E that is devoid of the capsid signal sequence and not to be secreted as a negative control. The *trans*-complemented particles (ZIKV_TCPs_) in the supernatants were used to infect naïve Vero cells (Figure 1(c)). The ZIKV_TCP_ infection was assessed by determining the luciferase activity at various time points post infection. For the ZIKV_TCP_ generated by CprM-E, there was obvious infection signal in the infected cells, as evidenced by amplification of sGluc signal. In contrast, for the ZIKV_TCP_ generated by prM-E, no infection signal was observed in the infected cells (Figure 1(d)). The ZIKV_TCP_ infection was prevented by pretreatment with bafilomycin and NHCl_4_, inhibitors for flavivirus entry [33,48], in a dose-dependent manner (Figure 1(e–f)) without obvious cytotoxicity (Supplementary Figure 1(a)). Taken together, we successfully generated a ZIKV *trans*-complemented particle system that mimics viral infection.

To facilitate detection of E, we inserted an HA tag or Flag tag into a tolerant site after the residue 147 of E as previous reported [36]. The insertion of the tag had no detrimental effect on the exposure of the E protein epitope and the neutralization sensitivity of tagged viruses by D1-4G2-4-15 (4G2) antibody which was a pan-flavivirus antibody that overlapped the E protein fusion loop [36]. We first generated infectious clones of C7-E^HA^ and C7-E^Flag^ (Supplementary Figure 1(b)). The C7-E^HA^ and C7-E^Flag^ virus replicated similarly to C7 virus in Vero cells, as evidenced by comparable viral protein levels in the infected cells (Supplementary Figure 1(c)). The expression of HA and Flag tags was verified by Western blotting (Supplementary Figure 1(c)). The C7-E^HA^ and C7-E^Flag^ virus also plaqued similarly to C7 in Vero cells (Supplementary Figure 1(d)). In the following studies, we chose the E^HA^ and generated ZIKV_TCP_ using CprM-E^HA^ (ZIKV_TCP_^HA^) as the method described above. Viral replication in the ZIKV_TCP_- and the ZIKV_TCP_^HA^-infected cells was examined by Western blotting analysis of the cell lysate and measuring the luciferase activity in the supernatants of the infected cells. There were comparable NS5 and E levels in the infected cells and comparable luciferase activity in the supernatants (Supplementary Figure 1(e) and Figure 1(g)), suggesting that ZIKV_TCP_^HA^ enters similarly to ZIKV_TCP._ The entry of ZIKV_TCP_^HA^ was also sensitive to bafilomycin A1 and NHCl_4_ (Supplementary Figure 1(f–g)). To further confirm that the insertion of the HA tag does not affect the virus assembly and entry, we transfected the same amount of prME or prME^HA^ into sgZIKV-sGluc-Vero cells, and the ZIKV_TCP_^HA^ exhibited similar egress and cellular binding as the ZIKV_TCP_ (Supplementary Figure 1(h–i)). Taken together, we generated a ZIKV *trans*-complemented particle system with HA-tagged E protein.

### Assembly and infectivity of ZIKV_TCP_^HA^ bearing E mutants

**Figure 2.**
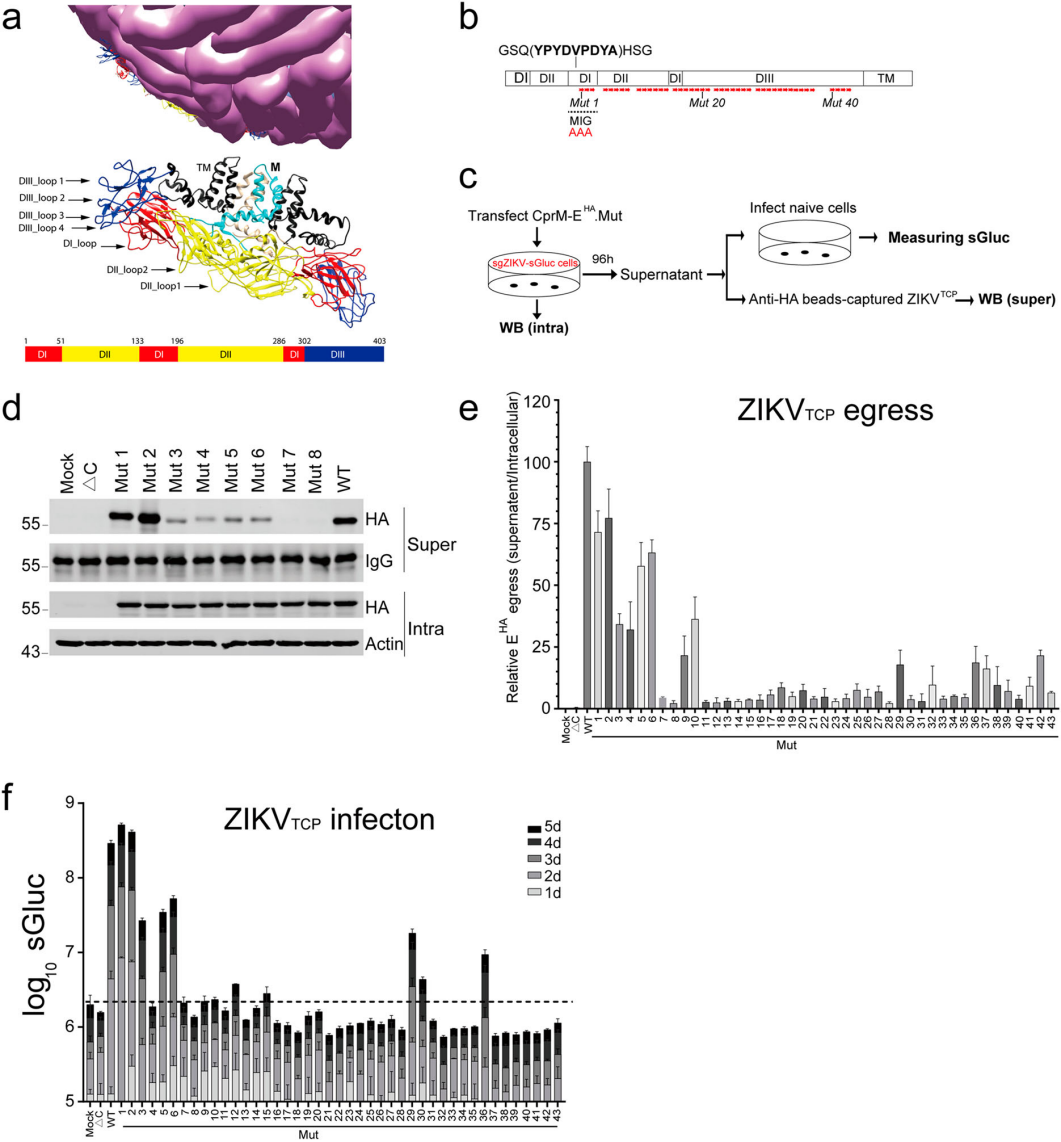
Assembly and infectivity of ZIKV_TCP_ bearing E mutants. (a) Structure of the mature ZIKV virion (PDB, 5iz7). The Loop regions (arrows) selected in this study and the schematic of ZIKV E domains are shown. (b) Schematic of ZIKV E mutagenesis. Three-amino acid mutations (Ala-Ala-Ala) (Red stars) are introduced in the indicated regions. The HA tag is inserted in the DI as indicated. (c) Schematic of the experimental design for D to F. Plasmids expressing CprM-E^HA^ (WT), prM-E (ΔC) and CprM-E^HA^-mutants were transfected into Vero-sgZIKV-sGluc cells, respectively. At 4 days post transfection, cell lysates (Intra) were harvested for Western blotting (WB) analysis. ZIKV_TCPs_ in the medium were captured by anti-HA beads (super) and analyzed by Western blotting. Alternatively, condition media were harvested and used to infect naïve Vero cells. At various time points, the luciferase activities in the supernatants of the infected cells were determined. (d) Cell lysates (Intra) and captured ZIKV_TCPs_ (Super) were analyzed by western blotting with the indicated antibodies. Representative pictures of Mut 1 to 8 are shown. The values to the left of the blots are molecular sizes in kilodaltons. (e) Summary of the Relative level of E^HA^ secreted into the supernatants to that in the intracellular cell lysates. Relative levels of E^HA^ egress of each mutants were calculated as (captured supernatant HA/Supernatant IgG)/ (Intracellular HA/Intracellular Actin) and further normalized to the WT. Data combined from two independent experiments are shown (mean ± SEM, *n* = 6). (f) Conditioned media containing ZIKV_TCPs_ were used to infect naïve Vero cells. The Gluc luciferase activities in the supernatants of the infected cells were determined at the indicated time points post infection. Mean values ± SDs are shown (*n* = 3).

By using the ZIKV_TCP_^HA^ system, we focused on the loop regions of the DIII that had been proposed to be involved in virus attachment and entry [26–29]. We also selected loop regions in the DI and DII that are on the surface of the virion (Figure 2(a)). We performed an alanine-scanning mutagenesis of these regions, with each mutant bearing three-alanine replacements (Figure 2(b) and Supplementary Table 2). We transfected the CprM-E^HA^ mutants or prM-E (ΔC) into the Vero-sgZIKV-sGluc cells as described above. The egress of the ZIKV_TCPs_^HA^ was assessed by measuring the intracellular expression of the E mutants and the released E in the supernatants after anti-HA immuno-capture. The infectivity of the released ZIKV_TCPs_^HA^ was determined by measuring the sGluc activity in the ZIKV_TCP_^HA-infected^ cells (Figure 2(c)).

Consistent to a previous study, the residues within the Mut 1 and Mut 2 are tolerant to exogenous insertion [36], Mut 1 and Mut 2 in the DI didn’t affect ZIKV_TCP_^HA^ assembly and infection. However, except for Mut 1 and Mut 2, all the mutants impaired or reduced the egress of the ZIKV_TCPs_^HA^, as determined by the relative levels of released E (Figure 2(d, e) and Supplementary Figure 2(a–f)). Accordingly, except for Mut 1 and Mut 2, all the mutants either impaired or abolished ZIKV_TCPs_^HA^ infection, evidenced by the luciferase activity in the infected cells (Figure 2(f)). Notably, Mut 4, Mut 9, Mut 10 and Mut 37 nearly completely abolished the infectivity of the ZIKV_TCPs_^HA^, albeit substantial E was released as evidenced by western blotting analysis (Figure 2(e–f) and Supplementary Figure 2(a, e)), suggesting that ZIKV_TCPs_ bearing these mutants fail to enter into cells (see below). The observation that most E mutants impaired ZIKV_TCPs_ egress is consistent with the previously proposed, participation of E in virion assembly [41].

### Impact of E mutations on prM expression

*Flavivirus* Capsid (C)-prM is processed by viral NS2B-3 protease in the cytosol and host signalase in the ER lumen. The signalase cleavage at C-prM is delayed until the viral protease cleaves the upstream of the prM signal sequence, which results in the production of prM and the capsid anchor (Ca) (Supplementary Figure 3(a)). Ca/pr cleavage is regulated by E expression and mutations in prM impair virion assembly [10,49–52]. As most E mutants impaired ZIKV_TCP_ egress, we examined the impact of these E mutants on prM-E processing. We transfected the CprM-E^HA^ mutants and prM-E^HA^ (ΔC) into Vero-sgZIKV-sGluc cells and examined the prM-E^HA^ processing by Western blotting analysis of E^HA^ and prM. Most E mutants were processed similarly as wild type (WT) E, except for Mut 42 (Supplementary Figure 3(c, d) and Supplementary Figure 4(a–c)). Mut 42 generated a band with smaller molecular weight than expected (Supplementary Figure 4(c)) (See below). Strikingly, Mut 4, Mut 7, Mut 8, Mut 11, Mut 13, Mut 14, Mut 15, Mut 17 Mut 18 and Mut 19 severely caused the reduction of prM expression (Supplementary Figures 3(c–e) and 4).

### Visualization of E trimer by a bioorthogonal system

At neutral pH in the ER lumen, the immature virion particle is composed of trimeric prM-E heterodimer. At acid pH in the trans-Golgi network (TGN), prM-E trimer undergoes conformation change into E homodimer, exposing the pr/M cleavage site and resulting in the furin-mediated pr-M cleavage and virion maturation [9,11,53].

**Figure 3.**
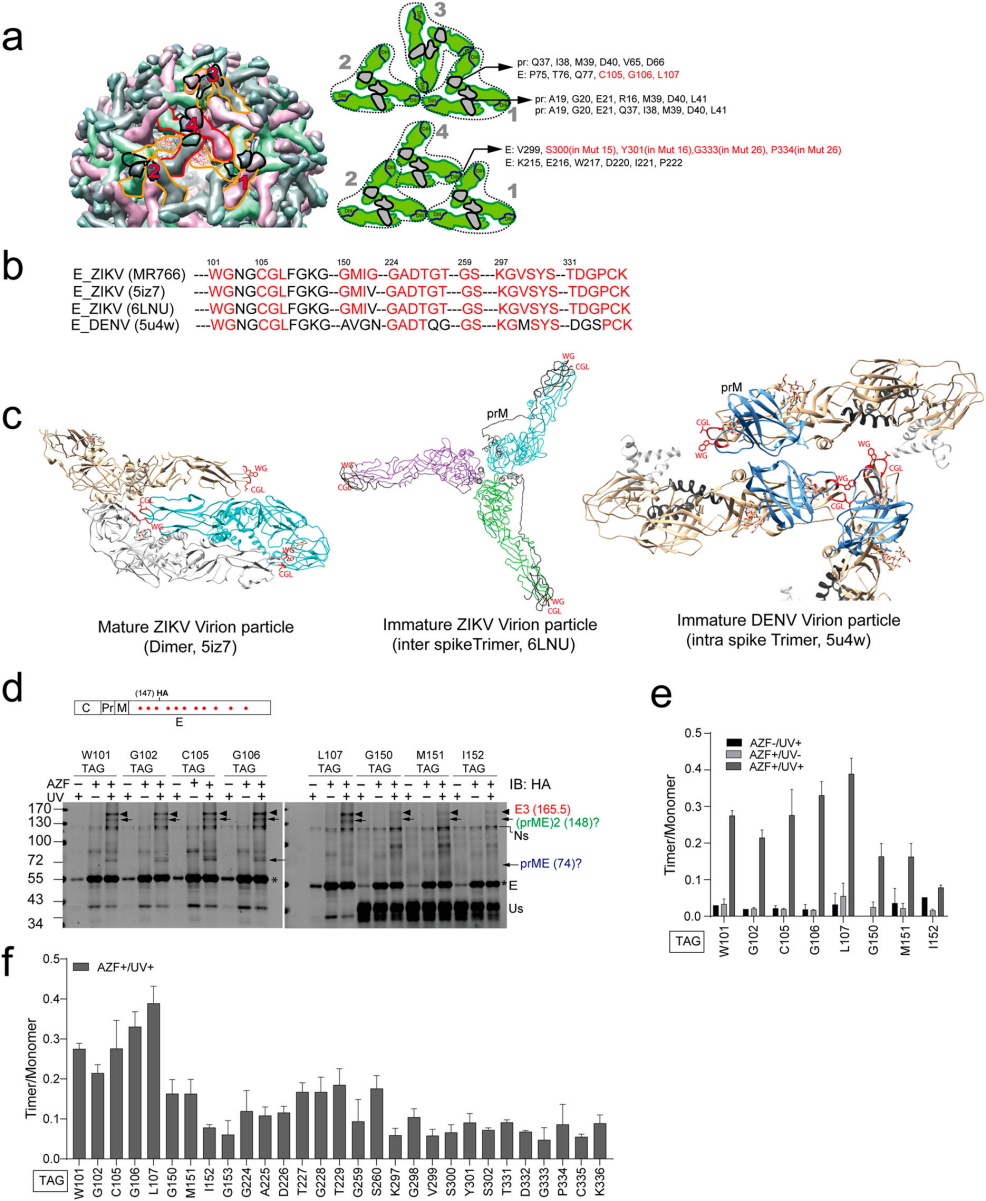
Visualization of E trimer by a bioorthogonal system. (a) Left: Structure of ZIKV immature virion (PDB, 6LNU). Four representative trimeric E-prM spikes are shown. Right: The E-prM interfaces within a spike and the E-E interface between two neighbouring spikes and the residues in the interfaces are shown. The residues selected in this study are indicated in red. (b) Alignment of the amino acids from different ZIKV strain and DENV within the interfaces. (c) Localizations of the selected residues (in *red*) in the interfaces in the structures. The prM in the immature virion is indicated. (d) The sgZIKV-sGluc-Vero cell line was cotransfected with plasmid pSVB.Yam, pcDNA.RS, and plasmids expressing CprM-E^HA^ with the TAG stop codon (red dots) introduced at the indicated residues. After UV-crosslinking, the cells were subjected to immunoprecipitation (IP) with anti-HA beads and the immunoprecipitated proteins were analyzed by Western blotting. The arrows indicate the E trimers (E3) with the expected molecular weight of 165.5 KD, the putative dimeric prME with the expected molecular weight of 148KD and the putative prME with the expected molecular weight of 74KD. The asterisks indicate the E monomers. Ns, nonspecific bands; Us, unspecified bands. The values to the left of the blots are molecular sizes in kilodaltons. (e) The abundances of the E trimers in (d) were quantified and the intensities of the E trimer were calculated as the ratio of E trimer/ E monomer. Mean values ± SD are shown (*n* = 3). (f) Summary of the results of the photo crosslinking results of all the E mutants. Mean values ± SD are shown (*n* = 3).

Some residues (G_298_V_299_S_300_Y_301_S_302_) in the assembly-dead mutants Mut15, Mut16, and residues (G_333_P_334_) in the Mut26 and Mut27 reside in the E-E interface within the prM-E trimer (Figure 2 and Figure 3(a–b)), suggesting a requirement for E timerization in virus assembly. Thus, we sought to explore the effect of the assembly-dead mutants on E multimerization during virion assembly. We recently used a bioorthogonal system [54] to visualize oligomerization and dimerization of hepatitis C virus proteins *in vivo* [38]. This bioorthogonal system uses the orthogonal suppressor tRNA/aminoacyl-tRNA synthetase (aaRS) pair for the photolabile unnatural amino acid (UAA) p-azido-L-phenylalanine (AZF). An amber codon (TAG) is introduced into the desired amino acid and AZF can be incorporated into the amber codon by the suppressor tRNA and aminoacyl tRNA synthetase. Under 365 nm UV irradiation, the AZF mediates photo crosslinking at specific residues at a distance range of 3–4 A°. Thus the photocrosslinking gives positional information of protein–protein interactions at the atomic level. The crosslinked protein complex was analyzed by denaturing SDS-PAGE and Western blotting (Supplementary Figure 6(a)).

First, we sought to identify residues that are able to mediate photocrosslinking of E dimer or trimer. Based on the ZIKV immature virion structure (PDB: 6LNU) (Figure 3(a)) [55], the ZIKV mature virion structure (PDB: 5IZ7) [56] and DENV immature virion structure (PDB: 5U4W) [57], we selected the equivalent ZIKV (MR766) E residues that are near the E dimer interfaces and prM-E trimer interfaces within the structures (Figure 3(b–c), Supplementary Figure 6(b) and Supplementary Figure 7). Residues that are near the E dimer (PDB: 5IZ7) interfaces include W_101_G_102_, G_150_M_151_I_152_G_153_, G_259_S_260_, G_224_A_225_D_226_T_227_G_228_T_229_ (Figure 3(b) and Supplementary Figure 6(b)); residues that near the prM-E interface within the trimeric spike (PDB: 6LNU) include C_105_G_106_L_107_; residues that are near the E-E interface between two neighbouring spikes include K_297_G_298_V_299_S_300_Y_301_S_302_ and T_331_D_332_G_333_P_334_C_335_K_336_ (Figure 3(a–c) and Supplementary Figure 6(b) and Supplementary Figure 7). We introduced TAG stop codons in these residues in CprM-E^HA^ and cotransfected these CprM-E^HA^ plasmids with plasmids expressing the suppressor tRNA and aminoacyl tRNA synthetase into Vero-sgZIKV-sGluc cells. In the presence of AZF, expression of the full-length E variants (E monomer) was restored (Figure 3(d) and Supplementary Figure 6(c–d)). Upon UV irradiation, specific irradiation-induced crosslinked bands with molecular weight equivalent to that of trimeric E^HA^ (E3, 165.5 kD) were observed. Obvious trimeric E crosslinking occurred via residues within the E dimer interface or the E trimers, including W_101_, G_102_, C_105_, G_106_, L_107_, G_224_, A_225_, D_226_, T_227_, G_228_, T_229_, G_259_, S_260_, K_297_, G_298_, V_229_, S_300_ and Y_301_ (arrows, Figure 3(d–f) and Supplementary Figure 6(c–d)) (see discussion). Residue L107 residing in the conserved flavivirus fusion loop, exhibited strongest trimeric E crosslink (Figure 3(f)). There were faint trimeric E crosslinked bands via the residues G_150_, M_151_, I_152_, G_153_, S_302_, T_331_, D_332_, G_333_, P_334_ and K_336_ (arrows, Supplementary Figure 6(c,d)). The reduced crosslinking by these residues is probably either due to the weak crosslink *per se* or due to the replacement of these residues by AZF that impairs E dimerization or trimerization. Notably, there were specific irradiation-induced crosslinked bands with molecular weight equivalent to that of dimeric prME (148 KD) and prME (74 KD) (arrows, Figure 3(d–f) and Supplementary Figure 6(c,d)). Putative dimeric prME crosslinking occurred via residues W_101_, G_102_, C_105_, G_106_, L_107_, G_224_, A_225_T_227_, G_228_, T_229_, G_259_ and S_260_ (arrows, Figure 3(d–f) and Supplementary Figure 6(c, d)). Putative prME crosslinking occurred via residues W_101_, G_102_, C_105_, and G_106_ (arrows, Figure 3(d–f) and Supplementary Figure 6(c, d)). Unfortunately, we couldn’t unambiguously detect these equivalent crosslinked bands with anti-prM antibodies (Supplementary Figure 6(e)), which might be due to the inadequate affinity of the antibodies. In addition, we didn’t detect crosslinked bands corresponding to E dimers (Figure 3 and Supplementary Figure 6) (see discussion).

We also monitored crosslinking via the residues C_105_, G_106_, L_107_ and M_151_ in Vero cells. In contrast to sgZIKV-Vero cells, in Vero cells, much less crosslinked E trimers were observed (Supplementary Figure 8(a, b)), which is likely due to the lack of Capsid-prM processing by NS3 and aberrant virion particle assembly.

### Impact of E mutations on E trimerization

**Figure 4.**
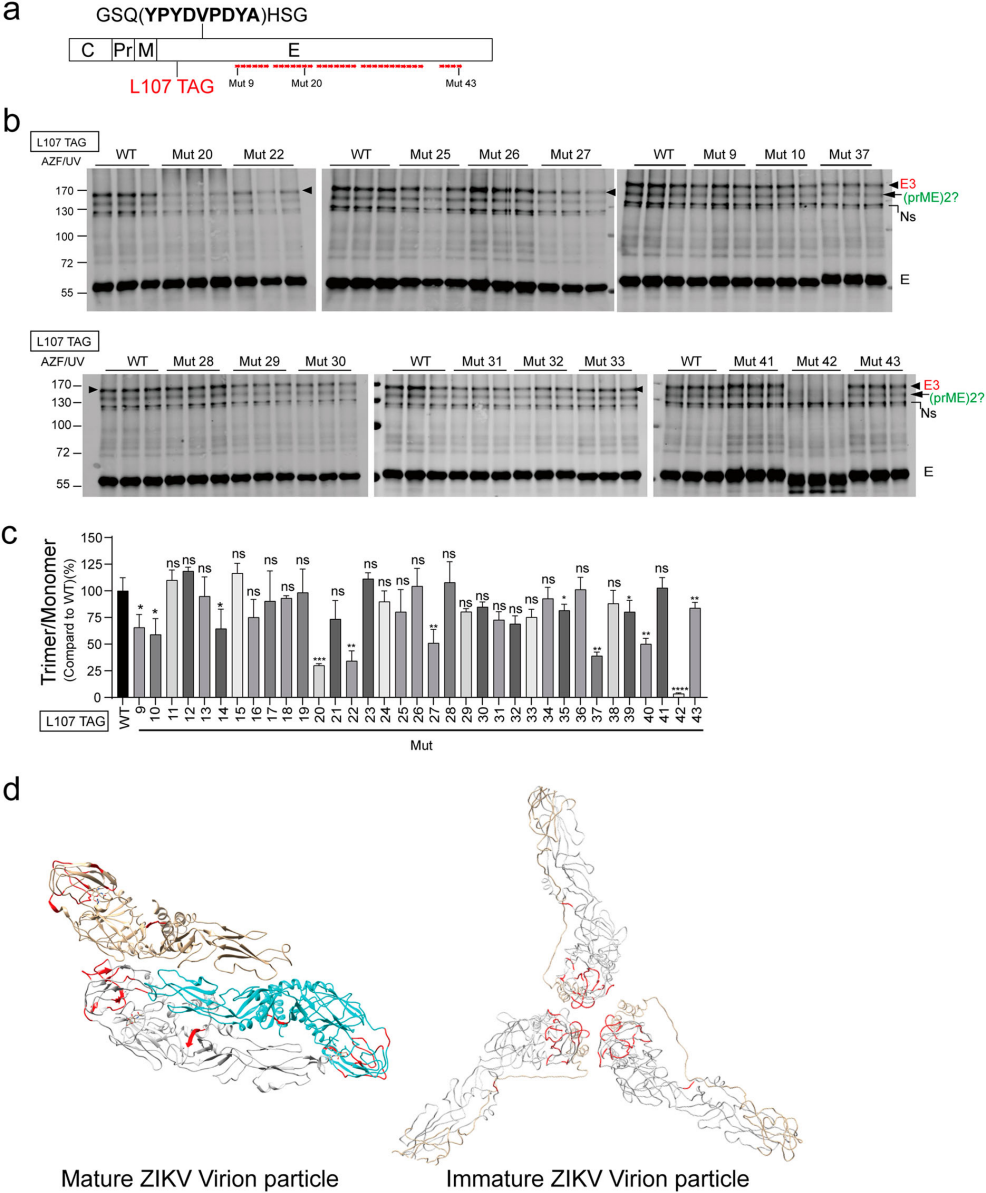
Impact of E mutations on E trimerization. (a) Schematic of HA-tagged E mutants with the TAG stop codon introduced at L107. (b) SgZIKV-sGluc-Vero cells were cotransfected with the plasmid pSVB.Yam, pcDNA.RS and the E mutant plasmids. After UV-crosslinking, the cell lysates were subjected to immunoprecipitation (IP) with anti-HA beads. Proteins were analyzed by Western blotting with anti-HA antibody. Representative Western blot of some mutants showing data from triplicate wells are shown. The values to the left of the blots are molecular sizes in kilodaltons. The E trimers (E3) and the putative dimeric prME are indicated. Ns, nonspecific bands. (c) The abundances of the E trimers in (b) were quantified. The relative intensities of the E trimers formed by the mutants were calculated (E trimer/ E monomer) and further normalized to WT. Mean values ± SDs are shown (*n* = 3). Statistical analysis was performed between WT and the mutants. (ns, not significant; *P < 0.05; **P < 0.01; ***P < 0.001; ****P < 0.0001; two-tailed, unpaired *t*-test). (d) Visualization of the equivalent residues (in *red*) of E mutants (Mut 20, Mut 22, Mut 27, Mut 37, Mut 40 and Mut 42) that impair E trimerization in mature virion (PDB, 5iz7) and immature virion (PDB, 6LNU) structures.

We then assessed the impact of the E mutant on E trimerization by monitoring their impact on the L107-mediated crosslinking. We introduced TAG in the L107 in the CprM-E^HA^ mutants (Figure 4(a) and Supplementary Table 1), and then cotransfected these CprM-E^HA^-L107TAG mutants, pSVB.Yam and pcDNA.RS into Vero-sgZIKV-sGluc cells. Upon photocrosslinking, mutants in DI and mutants in DII except Mut 9, Mut 10 and Mut 14 barely affected L107-mediated E trimerization (Figure 4(b,c) and Supplementary Figure 9(a, b)). However, mutants in DIII such as Mut 20, Mut 22, Mut 27, Mut 37, Mut 40 and Mut 42 significantly reduced E trimer formation over 50% percent and the putative prME dimer formation (Figure 4(b,c)). Strikingly, Mut 42, even though with a smaller molecular weight as expected (Supplementary Figure 4(c)), nearly exhibited no band of predicted trimeric form, suggesting that it abolished E trimerization (Figure 4(b,c)). Most residues corresponding to these mutants reside in the interface of the neighbouring spikes in the immature virion structure (Figure 4(d), in red).

### D389 is essential for E trimerization

**Figure 5.**
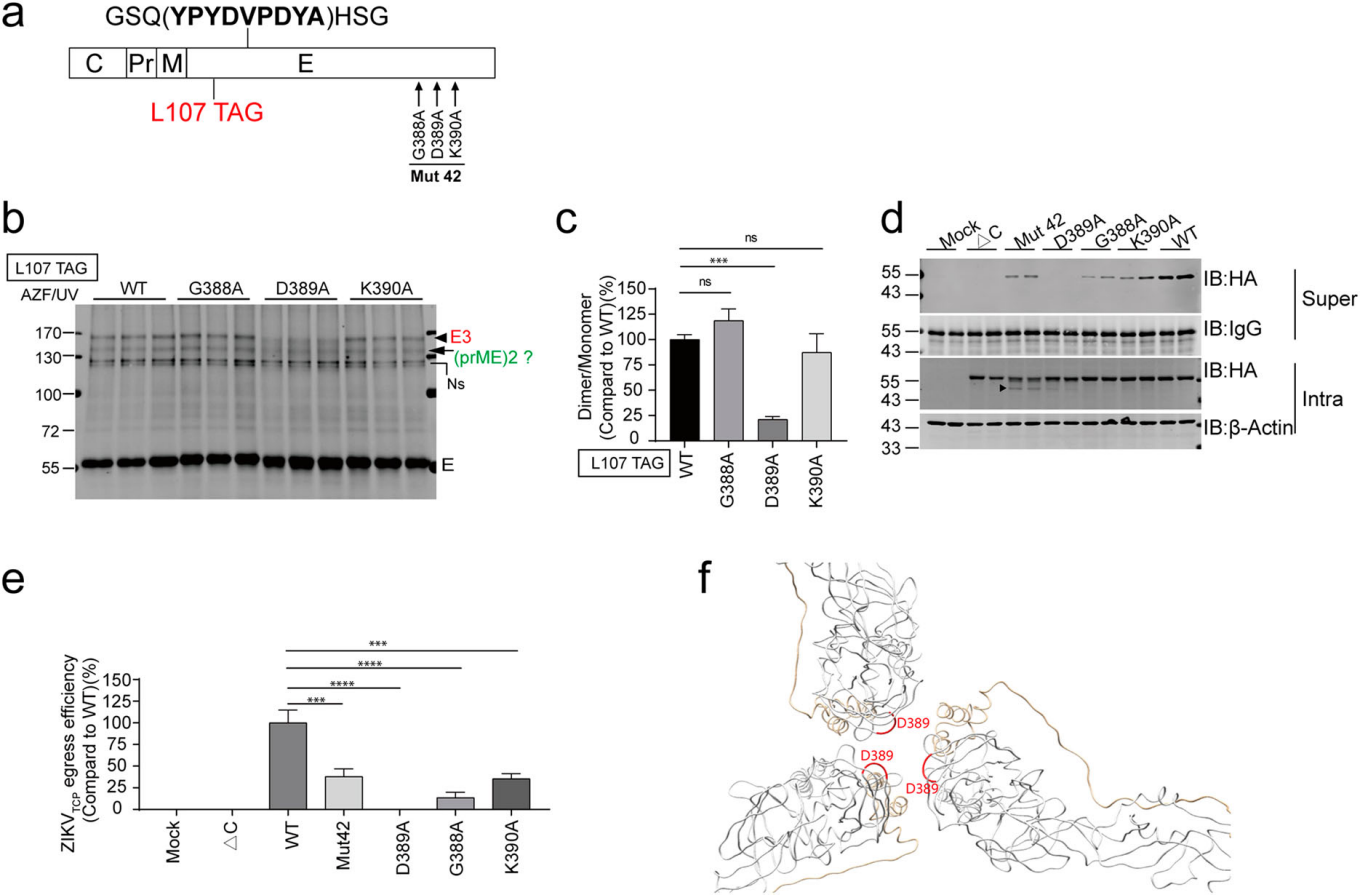
D389 is essential for E trimerization. (a) Schematic of HA-tagged E mutants with TAG stop codon introduced at L107. (b) SgZIKV-sGluc-Vero cells were cotransfected with the plasmid pSVB.Yam, pcDNA.RS and the E mutant plasmids. After UV-crosslinking, the cell lysates were subjected to immunoprecipitation (IP) with anti-HA beads. Proteins were analyzed by Western blotting with anti-HA antibody. The E trimers (E3) and the putative dimeric prME are indicated. Ns, nonspecific bands. The values to the left of the blots are molecular sizes in kilodaltons. (c) The abundances of the E trimers in (b) were quantified, and the relative intensities of the E trimers were calculated (E trimer/ E monomer) and then further normalized to WT. Mean values ± SDs are shown (*n* = 3). Statistical analysis was performed between L107 TAG-WT and the variants as indicated (ns, not significant; ***P < 0.001; two-tailed, unpaired *t*-test). (d) SgZIKV-sGluc-Vero cells were transfected with plasmids expressing CprM-E^HA^ (WT), prM-E^HA^ (ΔC) and CprM-E^HA^ mutants as indicated. At 4-day post transfection, ZIKV_TCPs_ in the supernatants (super) were captured by anti-HA beads and analyzed by Western blotting with anti-HA antibody. The cell lysates (Intra) were analyzed by Western blotting with the indicated antibodies. Representative Western blot with duplicated samples is shown. The values to the left of the blots are molecular sizes in kilodaltons. The arrow indicates a cleaved form of E. (e) The egress efficiency of ZIKV_TCPs_ in (d) were calculated as (captured supernatant HA/Supernatant IgG)/(Intracellular HA/Intracellular Actin). Data combined from two independent experiments are shown (mean ± SEM, *n* = 4). Statistical analysis was performed between WT and the mutants as indicated (ns, not significant; ***P < 0.001; ****P < 0.0001; two-tailed, unpaired *t*-test). (f) Visualization of the equivalent residues (in *red*) of D389 in the immature virion structure (PDB, 6LNU).

We further focused on the Mut 42 and did mutation on each residue to assess the effect on E trimerization (Figure 5(a)). D389A dramatically reduced E trimer formation whereas G388A and K390A did not (Figure 5(b,c)). Notably, as observed before, Mut 42 generated a smaller E fragment (Supplementary Figure 2(e), Supplementary Figure 4(c) and Figure 5(d)). Given that residues in Mut42 reside in the ER lumen, one possible explanation for the appearance of the 2 bands is that the AAA might mimic the C/prM cleavage site and lead to cleavage by signalase, which would be predicted to result in bands of 42.3 kilodaltons. Accordingly, there was a fragment with similar molecular weight of as expected when cleavage occurs at the residues in Mut 42 (arrow, Figure 5(d)). We also examined the effect of the mutants on virion egress (Figure 5(d,e)). ZIKV_TCPs_ generated by CprM-E^HA^ variants in the supernatants (super) were analyzed. The D389A mutation nearly completely abolished ZIKV_TCPs_ egress. Mut 42, G388A and K390A significantly reduced ZIKV_TCPs_ egress (see discussion). These results suggest a critical role of D389 in E trimerization and egress.

### Identification of residues important for virus entry

**Figure 6.**
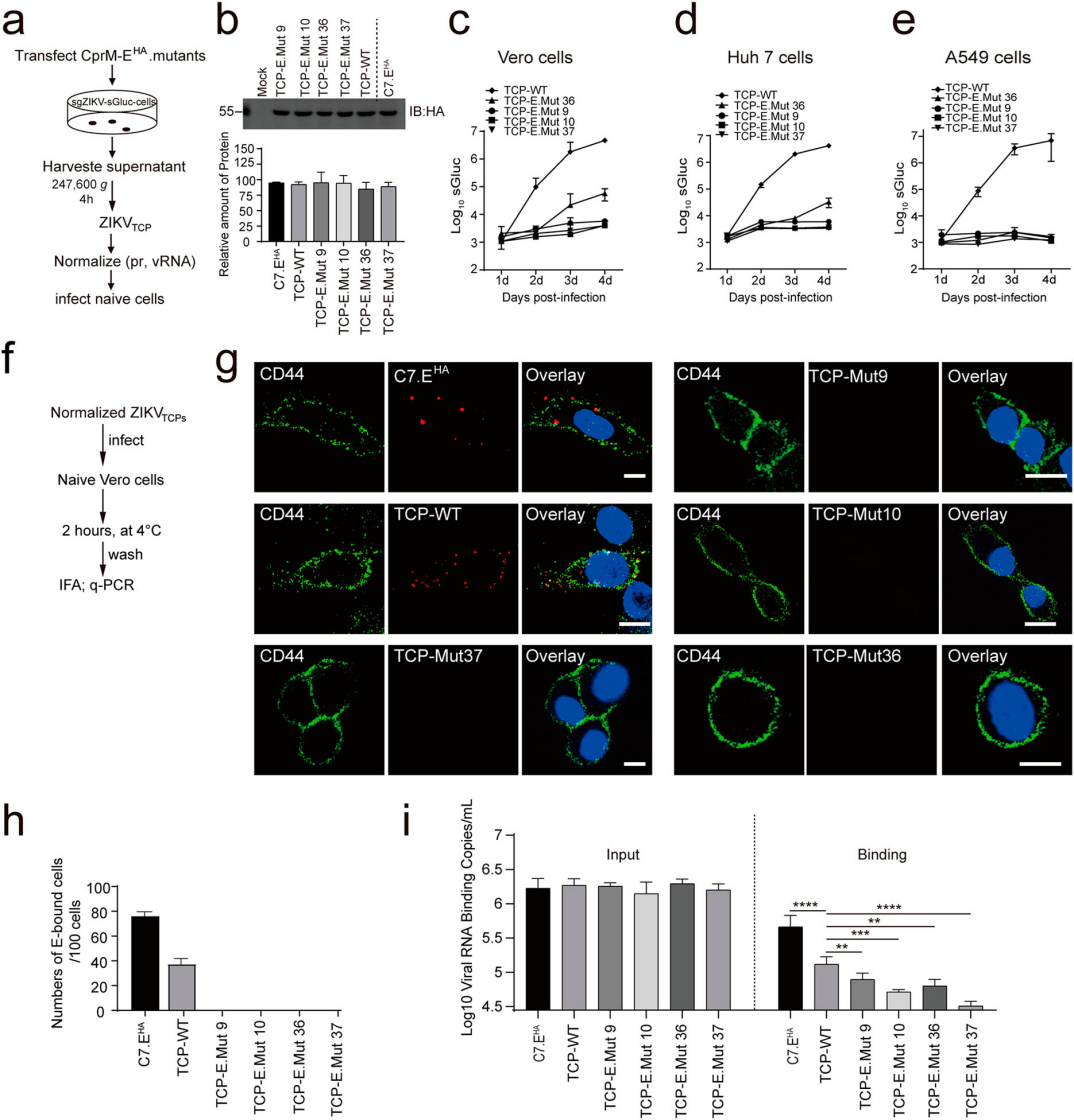
Identification of residues important for viral entry. (a, b) Concentration of ZIKV_TCP_. (a) Schematic of ZIKV_TCP_ concentration. Plasmids expressing HA-tagged E mutants Mut 9 (A268/L269A/E270A), Mut 10 (A271/E272A/M273A), Mut 36 (E366A/N367A/S368A) and Mut 37 (K369A/M370A/M371A) were transfected into sgZIKV-sGluc-Vero cells, respectively. ZIKV_TCPs_ in the supernatants were concentrated by ultracentrifugation through 20% sucrose cushion. ZIKV_TCPs_ were normalized to C7.E^HA^ by Western blotting analysis of viral E proteins. (b) Western blotting analysis of ZIKV_TCPs_ by anti-HA antibody (upper panel). A representative Western blot of triplicate samples is shown. Relative abundances of E proteins of each ZIKV_TCP_ to ZIKV_TCP_-WT were calculated (bottom panel). Mean values ± SDs are shown (*n* = 3). The values to the left of the blots are molecular sizes in kilodaltons. (c–e) Infection of Vero (c), Huh7 (d), A549 (e) by the concentrated ZIKV_TCPs_ in (b). At 1day post infection, the supernatants were removed, and the infected cells were washed and fresh media was added. The luciferase activities in the supernatants were measured at the indicated time points post infection. Mean values ± SDs are shown (*n* = 3). (f-i) Binding of ZIKV_TCPs_ to Vero cells. (f) Schematic of the experiment design. (g) Vero cells were infected with the normalized ZIKV_TCPs_ and C7.E^HA^ (MOI, 6) at 4°C for 2 h. Then the cells were washed three times with PBS and then fixed for immunostaining with anti-CD44 and anti-HA antibodies and AF488- and Cy3-conjugated 2nd antibody, respectively. The nucleus (blue) is stained by Hoechst 33342. Cells were observed with a confocal microscope. Representative images are shown. Scale bar, 10 μm. (h) The numbers of E-bound cells in (g) were quantified and plotted. Data combined from three independent experiments are shown (mean ± SEM, *n* = 6). (i) Vero cells were infected with the normalized ZIKV_TCPs_ and C7.E^HA^ (MOI, 6) at 4°C for 2 h. Then the cells were washed six times with PBS. The bound viral RNAs were extracted and quantified by q-PCR. Mean values ± SEMs from three independent experiments are shown (*n* = 9). Statistical analysis was performed as indicated (**P < 0.01, ***P < 0.001; ****P < 0.0001; two-tailed, unpaired *t*-test).

A group of mutants including Mut 9, Mut 10 and Mut 37 nearly abolished the infectivity of the ZIKV_TCPs_ without severely impairing the ZIKV_TCP_ egress (Figure 2(e,f)), suggesting effects of these mutants on viral entry. To assess the effect of these mutants on virus attachment to host cells, we first concentrated the ZIKV_TCPs_ generated by these mutants by centrifugation through a sucrose cushion. Then we normalized the ZIKV_TCPs_ against ZIKV virion to have equal amount of E proteins (Figure 6(a,b)). The normalized ZIKV_TCPs_ also had similar viral RNA genomes (Supplementary Figure 10(a)). We used the normalized ZIKV_TCPs_ to infect Vero, Huh7 and A549 cells. Viral infection was determined by measuring luciferase activity in the infected cells. Compared with ZIKV_TCP_-WT, the ZIKV_TCP_-mutants barely infected these cells, except that ZIKV_TCP_-Mut36 replicated to a limited level in Vero cells (Figure 6(c–e)). Then we examined the binding of the normalized ZIKV_TCPs_ to Vero cells. ZIKV_TCPs_ were incubated with Vero cells at 4°C for 2 h for attachment and then the bound virus was visualized by immunofluorescence staining against E and the bound viral RNAs were quantified by q-PCR (Figure 6(f)). The attachment of the ZIKV_TCP_-WT and C7.E^HA^ was evidenced by the colocalization of E proteins with a cell surface protein CD44 and the presence of cell-bound viral RNAs (Figure 6(g-i)). Notably, ZIKV_TCP_-WT had a lower binding efficiency than C7.E^HA^ as determined by quantification of cell-bound viral RNA (Figure 6(h-i)), which might be due to the existence of non-infectious ZIKV_TCPs_. In contrast to ZIKV_TCP_-WT and C7.E^HA^, ZIKV_TCP_-Mut 9, ZIKV_TCP_-Mut 10, ZIKV_TCP_-Mut 36, and ZIKV_TCP_-Mut 37 didn’t exhibit cellular attachment as no E staining signal was observed (Figure 6(g-h)). Accordingly, the cell-bound viral RNAs of ZIKV_TCP_-Mut 9, ZIKV_TCP_-Mut 10, ZIKV_TCP_-Mut 36, ZIKV_TCP_-Mut 37 were lowered about 3.5-8 fold, compared with ZIKV_TCP_-WT (Figure 6(i)). These results suggest that residues in Mut 9, Mut 10, Mut 36 and Mut 37 are important for virus attachment.

### Binding of ZIKV E-derived peptides to Vero cells

**Figure 7.**
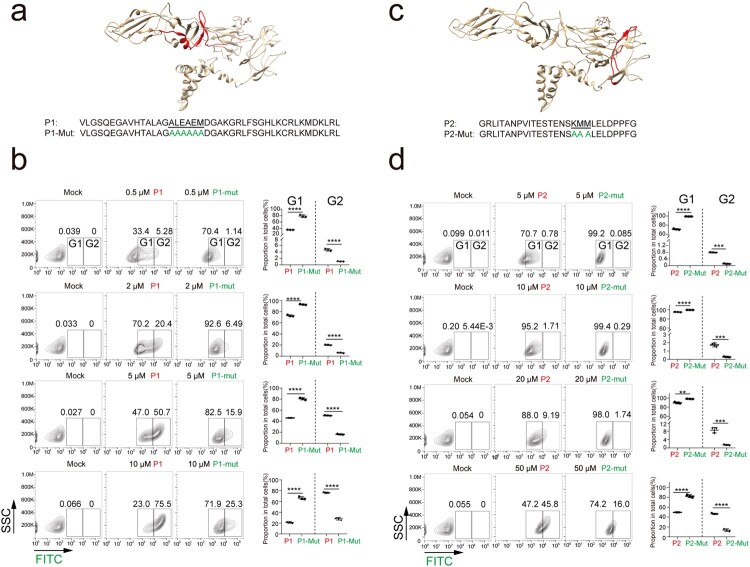
Binding of ZIKV E-derived peptides to Vero cells. (a) The P1 and P1-Mut sequences and the locations of the equivalent residues (in red) in the structure (PDB, 5i7z) are shown. The amino acids corresponding to Mut 9 and Mut 10 (green) are underlined. (b) Vero cells were incubated with PBS (Mock), FITC-conjugated peptides P1 and P1-Mut at different concentrations for 2 h at 4°C and then fixed and analyzed by flow cytometry as described in material and methods. (c) The P2 and P2-Mut sequences and the locations of the equivalent residues (highlighted) in the structure (PDB, 5i7z) are shown. The amino acids corresponding to Mut 37 (green) are underlined. (d) Vero cells were incubated with PBS (Mock), FITC-conjugated peptides P2 and P2-mut at different concentrations for 2 h at 4°C and then fixed and analyzed by flow cytometry. The SSC (y-axis) and FITC (x-axis) fluorescence intensities of the cells are shown. Gates to indicate binding of FITC-peptides were set on the mock-treated cells. Cell numbers of group 1 (G1) and group 2 (G2) were plotted. Mean values ± SDs are shown (*n* = 3). Statistical analysis was performed as indicated (**P < 0.01, ***P < 0.001; ****P < 0.0001; two-tailed, unpaired *t*-test.)

To further determine the role of the residues in Mut 9, Mut 10 and Mut 37 in ZIKV entry, we synthesized FITC-conjugated peptide P1 that contains the residues corresponding to Mut 9 and Mut 10 and peptide P2 that contains the residues corresponding to and Mut 37. We also synthesized FITC-conjugated peptide P1-Mut (FITC-P1-Mut) that contains the mutated residues in Mut 9 and Mut 10 and FITC-P2-Mut that contains the mutated residues in Mut 37, respectively (Figure 7(a, c) and Supplementary Figure 10(b, c)). The FITC-conjugated peptides were incubated with Vero cells for 2 h at 4°C and the binding of the peptides was quantified by flow cytometry. For control, we used peptides containing AAA mutations in the putative binding sites as control peptides (FITC-P1-Mut and FITC-P2-Mut) in the peptide binding experiments (Figure 7(a and c)). FITC-P1 and FITC-P1-Mut exhibited obvious binding at 0.5 μM whereas FITC-P2 and FITC-P2-Mut exhibited obvious binding at 5 μM (Figure 7(b and d) and Supplementary Figure 11(a)), suggesting that P1 has higher binding affinity than P2.

Notably, FITC-P1 and FITC-P1-Mut exhibited different binding patterns. There were two FITC-P1 bound cell populations: the group 1 (G1) with lower fluorescence intensity and the group 2 (G2) with higher fluorescence intensity. The G2 populations raised with the increase of peptide concentration, with significantly higher populations in the FITC-P1-bonund cells than that in the FITC-P1-Mut-bonund cells (Figure 7(b) and Supplementary Figure 11(b)), suggesting that the peptide FITC-P1-Mut with the mutated residues exhibited a lower binding affinity. Similarly, the G2 populations in the FITC-P2-bonund cells was higher than that in the FITC-P2-Mut-bonud cells (Figure 7(d) and Supplementary Figure 11(b)). These data indicate that Mut 9, Mut 10 and Mut 37 reduced the peptide binding to cell surface and residues in these mutants are important for cellular attachment.

### ZIKV E-derived peptides compete for ZIKV binding and infection

**Figure 8.**
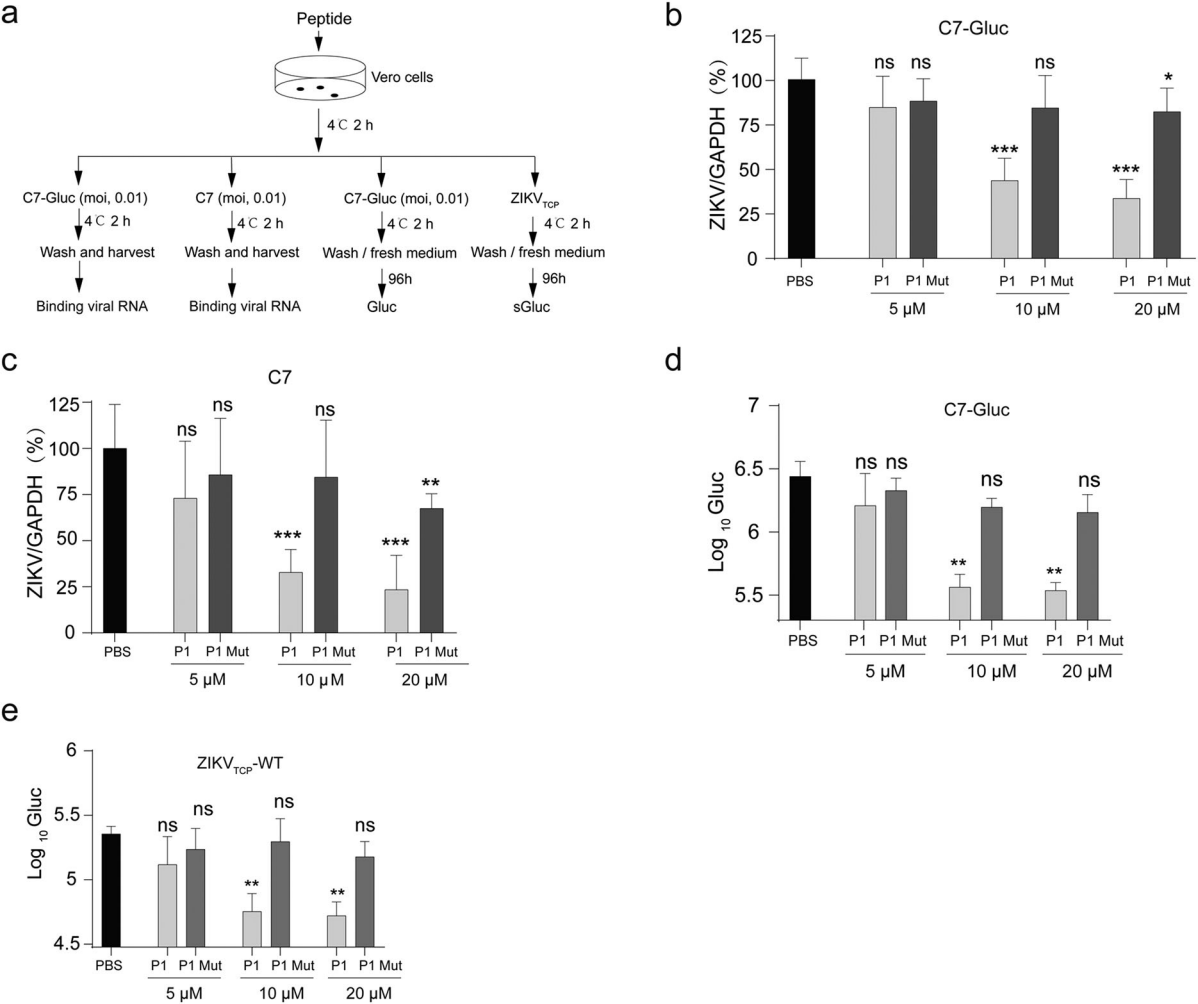
ZIKV E-derived peptides compete for ZIKV binding and infection. (a) Schematic of the experiment design. Vero cells were first incubated with various concentrations of P1 or P1-mut peptides as indicated, and then infected with C7-Gluc、C7 or ZIKV_TCP_ for 2 h at 4°C. After washing, the viral RNAs bound to cells were quantified or fresh media was added to the cells and the luciferase activity was determined at 4 days post infection. (b-c) Quantification of viral RNAs bound to cells. The bound C7-Gluc viral RNAs (b) and C7 viral RNAs (c) were extracted and quantified by q-PCR. Mean values ± SDs from two independent experiments are shown are shown (*n* = 6). Statistical analysis was performed as indicated (ns, not significant, *P < 0.05; ***P < 0.001, two-tailed, unpaired *t*-test). (d) Luciferase activity of the C7-Gluc infected cells. Mean values ± SDs are shown (*n* = 3). Statistical analysis was performed between the peptide-treated groups and the un-treated (PBS) group (ns, not significant, **P < 0.01, two-tailed, unpaired *t*-test). (e) Luciferase activity in the supernatants of the ZIKV_TCP_-infected cells. Mean values ± SDs are shown (*n* = 3). Statistical analysis was performed between the peptide-treated groups and the un-treated (PBS) group (ns, not significant, **P < 0.01, two-tailed, unpaired *t*-test).

We further explored the roles of the residues in these mutants in virus entry by wild-type Zika virus C7 and ZIKV_TCP_. We also used a ZIKV reporter virus C7-Gluc. Our previous study has showed that wild-type Zika virus C7 and ZIKV reporter virus C7-Gluc exhibited similar sensitivity to IFN [37]. C7-Gluc also had a similar growth curves and plaque morphology as wild-type virus C7 (Supplementary Figure 1(j-k)). The ZIKV *trans*-complemented particle system we constructed also had similar cell infectivity to C7-Gluc (Supplementary Figure 1(l-m)). As the P1 exhibited higher binding affinity to Vero cells, then we used P1 peptide to compete virus for cellular attachment and viral infection. We incubated Vero cells with various concentrations of P1 and P1-Mut peptides, and then infected the cells with wild-type Zika virus C7 and ZIKV reporter virus C7-Gluc at 4°C for 2 h. The cellular attachment was evaluated by quantifying the bound viral RNAs (Figure 8(a)). At 10 μM, P1 but not P1-Mut significantly reduced virus binding (Figure 8(b,c)). Then we used P1 to compete virus and ZIKV_TCPs_ for infection. Vero cells were treated with various concentrations of P1 or P1-Mut and then infected with C7-Gluc or normalized ZIKV_TCPs_ (Figure 8(a)). At 10 and 20 μM, compared to control (PBS), P1 significantly reduced C7-Gluc infection, evidenced by about 10-fold reduction of luciferase activity. In contrast, P1-Mut didn’t reduce viral replication (Figure 8(d)). Similarly, P1 but not P1-Mut significantly reduced ZIKV_TCPs_ infection at 10 μM and 20 μM, evidenced by about 5-fold reduction of luciferase activity (Figure 8(e)). These results suggest an important role of residues in Mut 9 and Mut 10 in virus attachment and infection.

## Discussion

Numerous residues distributed in DI, DII, DIII have been reported to affect flavivirus attachment and entry [22]. Antibody-mediated neutralization studies point to residues in DIII or outside DIII as important residues in virus entry [35]. However, systematic study of the putative key residues involved in virus attachment and entry is lacking. Here by using a ZIKV *trans*-complemented particle system (Figure 1 and Supplementary Figure 1), we performed mutagenesis of the residues in the loop regions in DIII, DI and DII that protrude from the surface of the virion and characterized the phenotypes of these mutants.

Previous study has identified some E mutations abolished virus rescue in the reverse genetic systems by using deep mutational scanning, which is important for understanding its function, its susceptibility to immunity and its future evolution [41]. In our research, we found that except for the loop regions in DI, most mutants impaired the egress of the ZIKV_TCPs_ (Figure 2(d–f) and Supplementary Figures 2 and 5). Of these mutants, mutations in DII and the hinge region of DI and DIII impaired viral egress by affecting prM expression (Supplementary Figure 3, Supplementary Figure 12(a) and Supplementary Figure 4). E expression affects the rate of signalase cleavage of prM [10]. The E mutants might similarly affect CprM processing. The egress-deficient mutants also include the K281 (in Mut12) that has been reported to be ubiquitinated to facilitate virus entry [24].

Flaviviruses assemble immature virions in the ER lumen. The immature virion particles contain trimeric prM-E heterodimers. In the acid environment in the trans-Gogi, the prM-E trimer undergoes conformation change, resulted in E dimer formation and exposure of the pr/M cleavage site [9]. Residues in some of the assembly-dead mutants (Mut15, Mut16, Mut26, Mut27) reside in the E-E interface within the E trimer (Figure 2 and Figure 3(a,b)), suggesting a requirement for E trimerization in virion assembly. To explore if the assembly-dead mutants affect the E dimerization or trimerization of prM-E, we employed a bioorthogonal system that could incorporate the photolabile unnatural amino acid AZF into specific E residues to photocrosslink multimeric E within cells (Supplementary Figure 6(a)).

First, we identified residues near the E dimer interfaces (W_101_G_102_, T_227_ G_228_T_229_, G_259_S_260_) in the mature virion and residues near the prM-E interface within the trimeric spike (C_105_G_106_L_107_) in the immature virion to mediate photocrosslinking of E trimers and putative prME dimers (Figure 3 and Supplementary Figure 6). The photocrosslinked E trimers and putative prME dimers by these residues near the E dimer interfaces in the mature virion are unlikely crosslinked solely by the matured E dimer or the immature trimer, as based on the structures of the ZIKV mature virion (5iz7) and DENV intra spike trimer (5U4W), these residues could not crosslink into a trimer (Figure 3(a, c) and Supplementary Figure 6(b)). Rather, the crosslinked E trimers are probably a mixture of a mature E-dimer crosslinked to another E in an immature E-trimer. Given that the flavivirus virions have been proposed to undergo reversible structural changes [58], a mixture of mature virion and immature virion probably exists within the cells. We didn’t detect crosslinked bands with molecular weight equivalent to that of dimeric E in the intracellular samples (Figure 3 and supplementary Figure 6). As prM-E dimerization occurs prior to E-E dimerization, and the putative prM-E dimers are detected (Figure 3), the absence of E dimer may imply that the E are tightly engaged with prM-E or E trimers within the cell. Similarly, the crosslinked E trimers by the residues (C_105_G_106_L_107_) near the prM-E interface within the trimeric spike in immature virions are probably a mixture of mature E-dimer and immature E-trimer, as these residues are also near the E dimer interface in the mature virion (Figure 3(c)). Nevertheless, the crosslinking of E trimers would reflect the trimerization of E *in vivo.* We then assessed the assembly-dead E mutants on E crosslinking via the residue L107 which is part of the flavivirus conserved fusion loop. Previous flavivirus studies have demonstrated the hinge regions of the E protein that are critical for viral replication and fusion involved in the transition from dimer to trimer [59]. Upon photocrosslinking, we found that mutants Mut 9, Mut 10 and Mut 14, that are in the hinge regions of the E protein affected L107-mediated E trimerization.

Strikingly, we found that numerous mutations in the DIII impaired E trimerization (Figure 4, Supplementary Figure 9 and Supplementary Figure 12(b)) and the D389A mutation nearly abolished E trimerization and viral egress (Figure 5). These residues reside in the E-E interfaces in the neighbouring spikes in the immature virion (Figure 4(d) and Figure 5(f)). These data suggest a role of DIII in mediating the E-E interactions in the neighbouring spikes within the immature virion.

After receptor binding-mediated endocytosis, flavivirus membrane fusion and uncoating take place in the late endosome, accompanied by rearrangement of homodimeric E to a fusion-competent homotrimers [60]. Antibodies targeting the cellular attachment and the post-fusion process all would neutralize viral infection. Direct evidences of key E residues that mediate receptor binding are lacking. In this study, we found that several E mutants, including Mut 9, Mut 10, Mut 36 and Mut 37, nearly abolished viral infectivity, albeit with normal prM expression and substantial E released, suggesting effects of these mutants on viral attachment (Figure 2(e–f), Supplementary Figure 3 and Supplementary Figure 12(b, c)). ZIKV_TCPs_ bearing these mutants have defects in cellular attachment, evidenced by reduced immunostaining of the bound virion and virion-associated viral genome (Figure 6). These results suggest critical roles of these residues in cellular attachment. It should be noted that some mutants, such as mutant 29, 32, 36, 39, 41 and 42, that impaired virus assembly as mutant 4, 9, 10 and 37 (Figure 2(e–f), Supplementary Figure 2). Unfortunately, we couldn’t get the concentrated ZIKV_TCPs_ by the means we used here due to their extremely low abundances. Further, FITC-conjugated peptide (P1) that contains the residues corresponding to the Mut 9, Mut 10 and peptide (P2) that contains residues corresponding to the Mut 37 bound to Vero cells, whilst P2 exhibited less binding than P1 (Figure 7 and Supplementary Figure 10(b–c)). P1 significantly competed with ZIKV for binding of ZIKV and infections of ZIKV or ZIKV_TCP_ at a concentration of 10 μM (Figure 8). These data indicate that residues A_268_L_269_E_270_A_271_E_272_M_273_ (Mut9 and Mut10) play an important role in virus entry at the attachment step, although we can’t exclude their also having roles at a post-attachment step. Given the Mut37 dramatically reduced attachment, as determined by quantification of the bound viral RNA (Figure 6(i)), the reduced binding of P2 might be either due to a lower binding affinity of P2 or inefficient folding of P2.

DIII protrudes above the viral surface [26] and has been proposed to act as receptor-binding domain based on binding assays [27–29]. Structure of the post-fusion E trimers implies conformational and positional changes of the E dimers on the viral surface during virus entry, including positional movement of DII from anti-parallel to parallel. The driving forces for the conformational and positional changes would be either receptor binding or acidification in endosome [26]. We propose that the DII residues A_268_L_269_E_270_A_271_E_272_M_273_-mediated receptor binding might contribute to initiation of these conformation changes. Notably, anti-Flag antibody blocks a step after cell attachment of ZIKV that bears the Flag insertion after R279 and L280 [36], which are nearing the residue A_268_L_269_E_270_A_271_E_272_M_273._ Antibodies against these residues can be used to further characterize the roles of these residues in virus entry in the future studies.

In summary, in a ZIKV *trans*-complemented particle (ZIKV_TCP_) system, we performed mutagenesis of the loop regions in the envelope (E) protein ectodomains, for their roles in viral egress and entry. We identified key resides that affect viral egress and entry. We unveiled the requirement for DIII in E trimerization and virus assembly and uncovered resides (268-273aa in DII) that play important role in virus attachment. Our findings advance understanding of the mechanism of flavivirus entry and egress.

## Supplementary Material

Supplemental Material
