# Supplementary material for: Identification and characterization of key residues in Zika virus envelope protein for virus assembly and entry
Source: Emerg Microbes Infect. 2022 Jun 10;11(1):1604–20. doi: 10.1080/22221751.2022.2082888 (PMC9196690; doi:10.1080/22221751.2022.2082888)
Supplement: Supplemental Material [file TEMI_A_2082888_SM5429.zip › Supplementary materials_EMI_224110199.docx]

**Supplementary material and methods**

**Cells**

### The human hepatoma cell line (Huh 7), Human embryonic kidney cell line (HEK293T) and African green monkey kidney cell line (Vero) were purchased from the National Collection of Authenticated Cell Cultures, Shanghai, China ([www.cellbank.org.cn](http://www.cellbank.org.cn/)). HEK293T were maintained in Dulbecco modified medium supplemented with 10% FBS (Gibco catalog no. 10099-141C), 1% penicillin/streptomycin (Biological Industries) and 25 mM HEPES (Gibco). Huh7 cells and Vero were maintained in Dulbecco modified medium supplemented with 10% FBS (Biological Industries catalog no. 04-001-1), 1% penicillin/streptomycin (Biological Industries) and 25 mM HEPES (Gibco). To generate sgZIKV-sGluc replicon cells, Vero cells were transfected with *in-vitro*-transcribed sgZIKV-sGluc RNA and then cultured with medium supplemented with 5 μg/ml[Blasticidin](https://www.so.com/link?m=b4wN1qh6L7REHutp8Pe5GuQzMBiLn3OXoBJdeZ3KiEEblO6jv0V8rVImsEwxT7bkaFktpFZrx0oNfSxll7HJY16lR7JAf80zQhme6J0emn6eI9BOUBGAAw8bsMDI7lCkPDTYKBjviqjihnHtz4Tw6HqDNAYTZC34RVR9e0ckSfSvxp7DBvqLm1w==). The surviving cells were pooled and maintained in conditioned medium with 5 μg/ml [Blasticidin](https://www.so.com/link?m=b4wN1qh6L7REHutp8Pe5GuQzMBiLn3OXoBJdeZ3KiEEblO6jv0V8rVImsEwxT7bkaFktpFZrx0oNfSxll7HJY16lR7JAf80zQhme6J0emn6eI9BOUBGAAw8bsMDI7lCkPDTYKBjviqjihnHtz4Tw6HqDNAYTZC34RVR9e0ckSfSvxp7DBvqLm1w==).

**Antibodies and Chemicals**

Anti-HA mouse monoclonal antibody (Abcam, ab130275) and anti-HA rabbit monoclonal antibody (Cell signaling technology, C29F4) were used in Western blotting at 1:1000 dilution and in immunostaining at 1:300 dilution, respectively. Anti-β-Actin antibody (A1978; Sigma) was used at 1:5,000 dilution in Western blotting. Anti-ZIKV NS5 antibody (GTX133312; GeneTex), anti-ZIKV NS3 antibody (GTX133309; GeneTex) and anti-ZIKV E (GTX133314; GeneTex) were used in Western blotting at 1:1000 dilution. Anti-ZIKV prM (GTX133305; GeneTex) were used in Western blotting at 1:300 dilution. Anti-CD44 antibody (Abcam, ab130275) was used in immunostaining at 1:300 dilution. Goat-anti-mouse IRDye 800CW secondary antibody (licor; 926-32210) and goat-anti-rabbit IRDye 800CW secondary antibody (licor; 926-32211) were used in Western blotting at 1:10000 dilution.

NHCl_4_ was dissolved in DPBS to get a 100 mM stock. Bafilomycin A1 (B1793; Sigma) was dissolved in DPBS to get a 0.1μM stock. MG132 (M7449; Sigma) was dissolved in DPBS to get a 10 μM stock. 4-Azido-L-phenylalanine (CAS No. 33173-53-4) was purchased by MedChemExpress (HY-16714) and was dissolved in pre-warmed cell culture medium at a final concentration of 0.5 mM before use.

***In vitro* transcription**

To generate C7.E^HA^ and C7.E.^Flag^ virus, the C7.E^HA^ and C7.E^Flag^ plasmids were linearized by AfeI (Fermentas, ER0321, fast digestion) digestion and purified by MinElute Gel Extraction Kit (Qiagen). DNAs were eluted in nuclease-free water and used as templates in *in vitro* transcription by mMESSAGE mMACHINE™ SP6 (Invitrogen, AM1340). The *in vitro*-transcribed RNAs were purified by RNeasy Mini Kit (Qiagen) and eluted in nuclease-free water.

**Reverse transcription (RT)-PCR.**

RNAs were purified by TRIzol extraction and reversely transcribed with PrimeScript RT reagent kit with gDNA Eraser (Perfect Real Time, TaKaRa, RR047A-1) according to the manufacturer’s instructions. The cDNA samples were subjected to real-time PCR (SYBR Premix Ex Taq Tli RNase H Plus, TaKaRa, TaKaRa, RR420) with the following primers for specific genes: GAPDH, (sense: 5-GGT ATC GTG GAA GGA CTC ATG A and (antisense: 5-ATG CCA GTG GCT TCC CGT TCA GC; ZIKV, (sense: 5-GGC GGTCAG TGG AGA TGA CTG C) and (antisense: 5-CCG GAT GCT CCA TCC TGCC). Serially diluted *in vitro*-transcribed ZIKV were used as standard templates.

**Western blot analysis**

Cells were lysed directly with 2×SDS loading buffer (100 mM Tris-HCl, pH 6.8, 20% glycerol, 4% SDS, 10% 2-mercaptoethanol, 0.02% bromophenol blue) and then boiled for 5 min. Samples were separated by SDS-PAGE and transferred to nitrocellulose membrane (Schleicher & Schuell BioScience). The membranes were incubated with blocking buffer (PBS, 5% milk, 0.05% Tween) for 2 hours. Blots were probed with different primary antibodies followed by secondary antibody. Protein bands were visualized by Odyssey CLx Imaging System. The protein bands were quantified by densitometry with ImageJ if necessary.

**Luciferase activity**

Supernatants were taken and mixed with equal volume of 2 x passive lysis buffer (Promega). Cells in 48-well plate were lysed in 60 μl of 1 × passive lysis buffer (Promega). Ten microliters of the lysates were mixed with 50 μl Renilla luciferase substrate (Promega) and the luciferase activity was measured by a GLOMX luminometer (Promega).

**Cell viability**

Cell viability was measured by Cell Counting Kit-8 (DOJINDO) according to the manufacturer’s protocol.

**Supplementary Figures**

**Supplementary Figure 1**


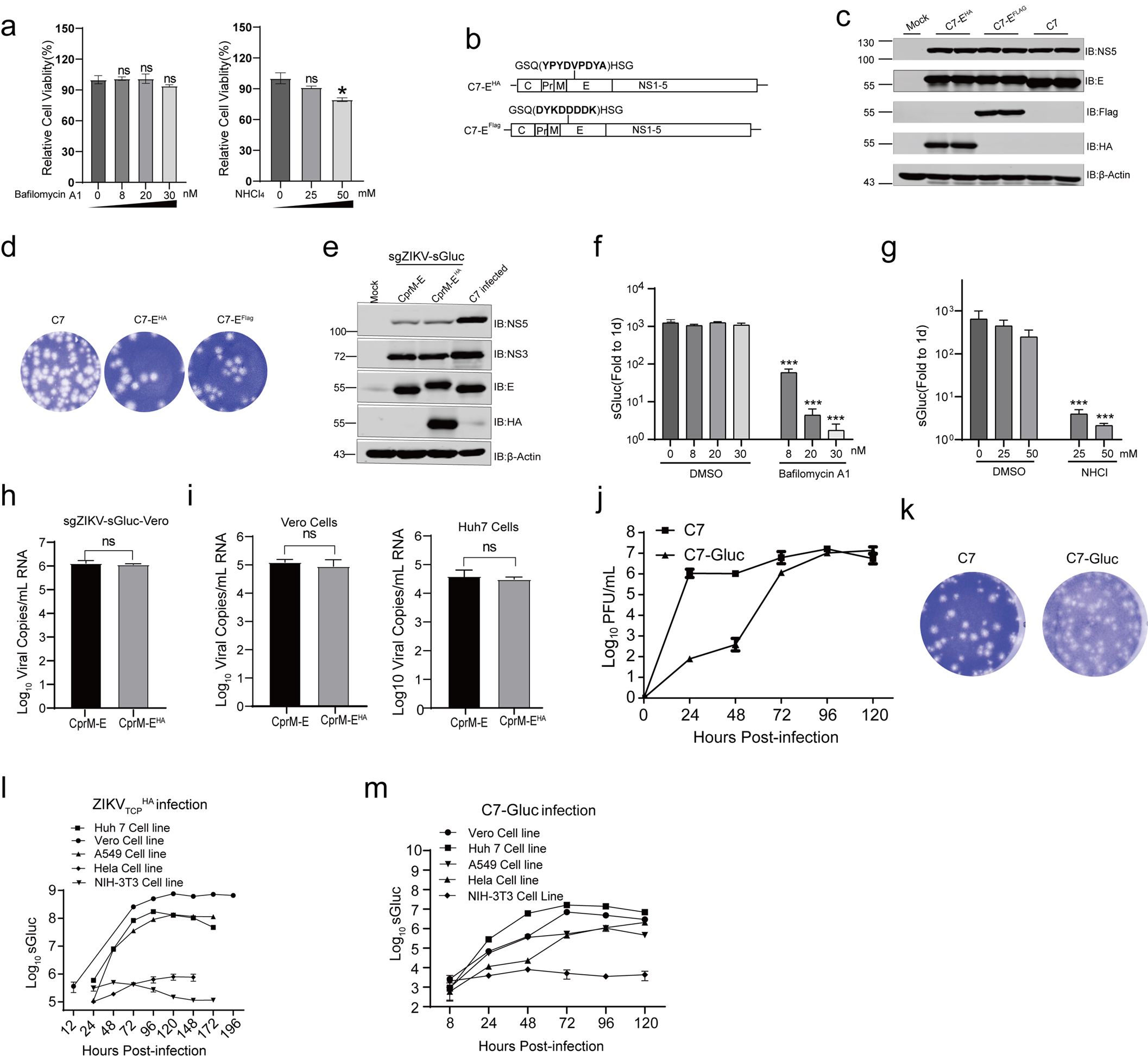


**Supplementary Figure 1. Generation of E-tagged ZIKV and ZIKV_TCP._** (a) Cell viability of Bafilomycin- and NHCl_4_-treated cells. Vero cells were infected with ZIKV_TCP_ for 1 day in the presence of various concentrations of Bafilomycin and NHCl_4_. Then the supernatants were removed, and fresh media was added with inhibitors. Three days later, cell viability was determined. Mean values ± SDs are shown (n = 3). Statistical analysis was performed between the treated groups and the control-treated (0) group. (ns, not significant, *P < 0.01; two-tailed, unpaired *t*-test). (b) Schematic diagram of C7-E^Flag^ and C7-E^HA^. The HA tag (YPYDVPDYA) and the Flag tag (DYKDDDDK) were inserted after the residue 147 of E protein. (c) Western blotting analysis of the ZIKV infected cells. Vero cells were infected with C7, C7-E^Flag^ and C7-E^HA^ at MOI of 1, respectively. Cells were harvested at 4 days post infection and cell lysates were analyzed by Western blotting with the indicated antibodies. Data for the samples from duplicate wells are shown. The values to the left of the blots are molecular sizes in kilodaltons. (d) Representative plaque morphology of C7, C7-E^Flag^ and C7-E^Flag^ harvested at 4 days post electroporation. (e) sgZIKV-sGluc-Vero cells were transfected with plasmids expressing CprM-E or CprM-E^HA^, and then cells were harvested at 2 days post transfection and analyzed by Western blotting with the indicated antibodies. Cell lysates at 2 days post infection of Vero cells infected with C7 at MOI of 1 were used as a control. The values to the left of the blots are molecular sizes in kilodaltons. (f and g) Inhibition of ZIKV_TCP_^HA^ by Bafilomycin and NHCl_4_. Vero cells were incubated with various concentrations of Bafilomycin (f) and NHCl_4_ (g) for 2 hours, and then infected with ZIKV_TCP_-E^HA^. The corresponding different concentrations of DMSO are used as controls. At 1day post infection, the cells were washed with PBS and fresh media was added. The luciferase activities in the supernatants were determined at 1 day and 4 days post infection. The relative luciferase activities at 4-day post infection to those at 1-day post infection are plotted. Mean values ± SDs are shown (n = 3). (h) sgZIKV-sGluc-Vero cells were transfected with plasmids expressing CprM-E or CprM-E^HA^, and then supernatant were harvested at 4 days post transfection, Viral RNA produced in the supernatant were extracted and quantified by q-PCR. (i) Vero and Huh 7 cells were infected with the supernatant in (h) at 4℃ for 2 hours. Then the cells were washed six times with PBS. The bound viral RNAs were extracted and quantified by q-PCR. Mean values ± SDs are shown (n = 3). (j and k) Growth curves of Zika virus C7 and C7-Gluc were made (j), and Representative plaque morphology of C7 and C7-Gluc were shown (k). Mean values ± SDs are shown (n = 3). (l and m) Different cell lines were infected with ZIKV_TCP_-E^HA^ (l) and C7-Gluc MoI=1 (m), respectively, and luciferase activities were determined at various time points. Mean values ± SDs are shown (n = 3).

**Supplementary Figure 2**


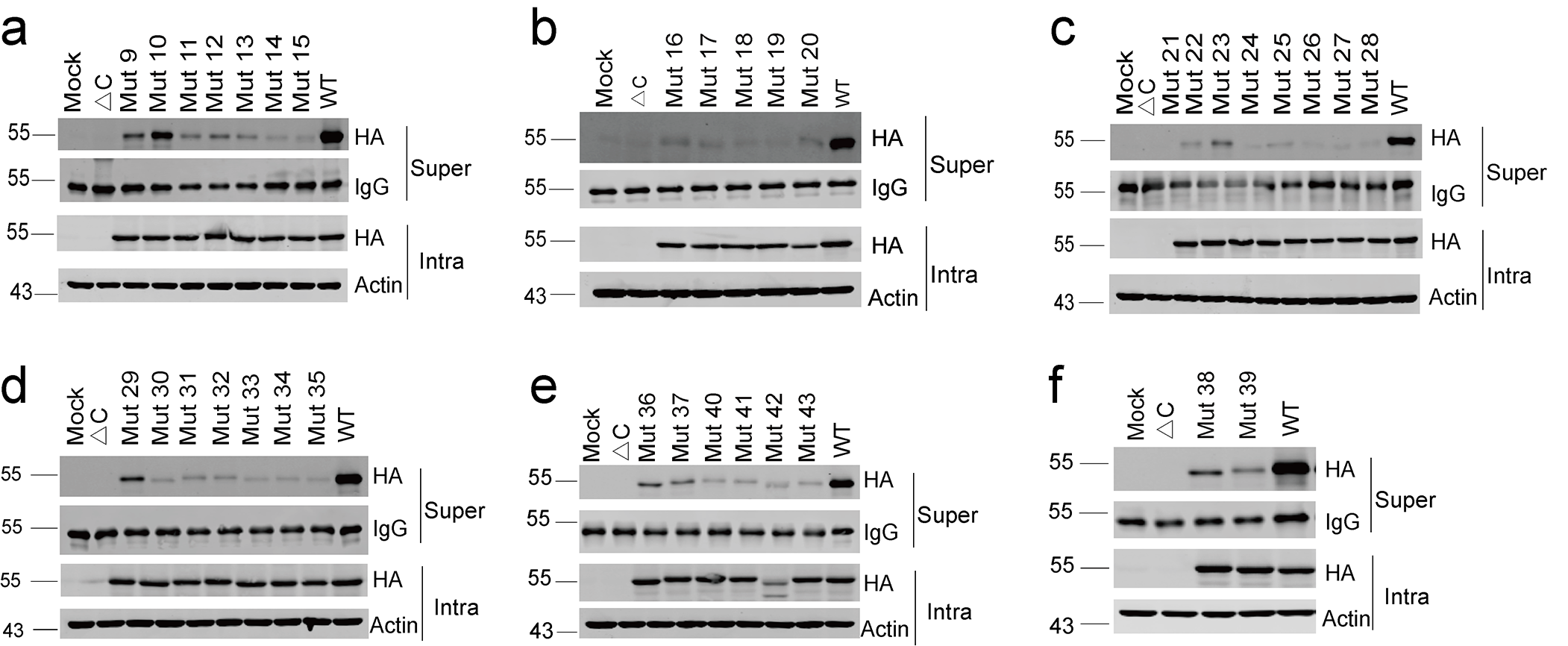


**Supplementary Figure 2. Assembly and infectivity of ZIKV_TCP_ bearing E mutants.** (a-f) Plasmids expressing CprM-E^HA^ (WT), prM-E (∆C) and CprM-E^HA^-mutants were transfected into Vero-sgZIKV-sGluc cells, respectively. At 4 days post transfection, cell lysates (Intra) were harvested for Western blotting analysis. ZIKV_TCP_ (super) were captured by anti-HA beads and analyzed by Western blotting with the indicated the antibodies. The values to the left of the blots are molecular sizes in kilodaltons.

**Supplementary Figure 3**


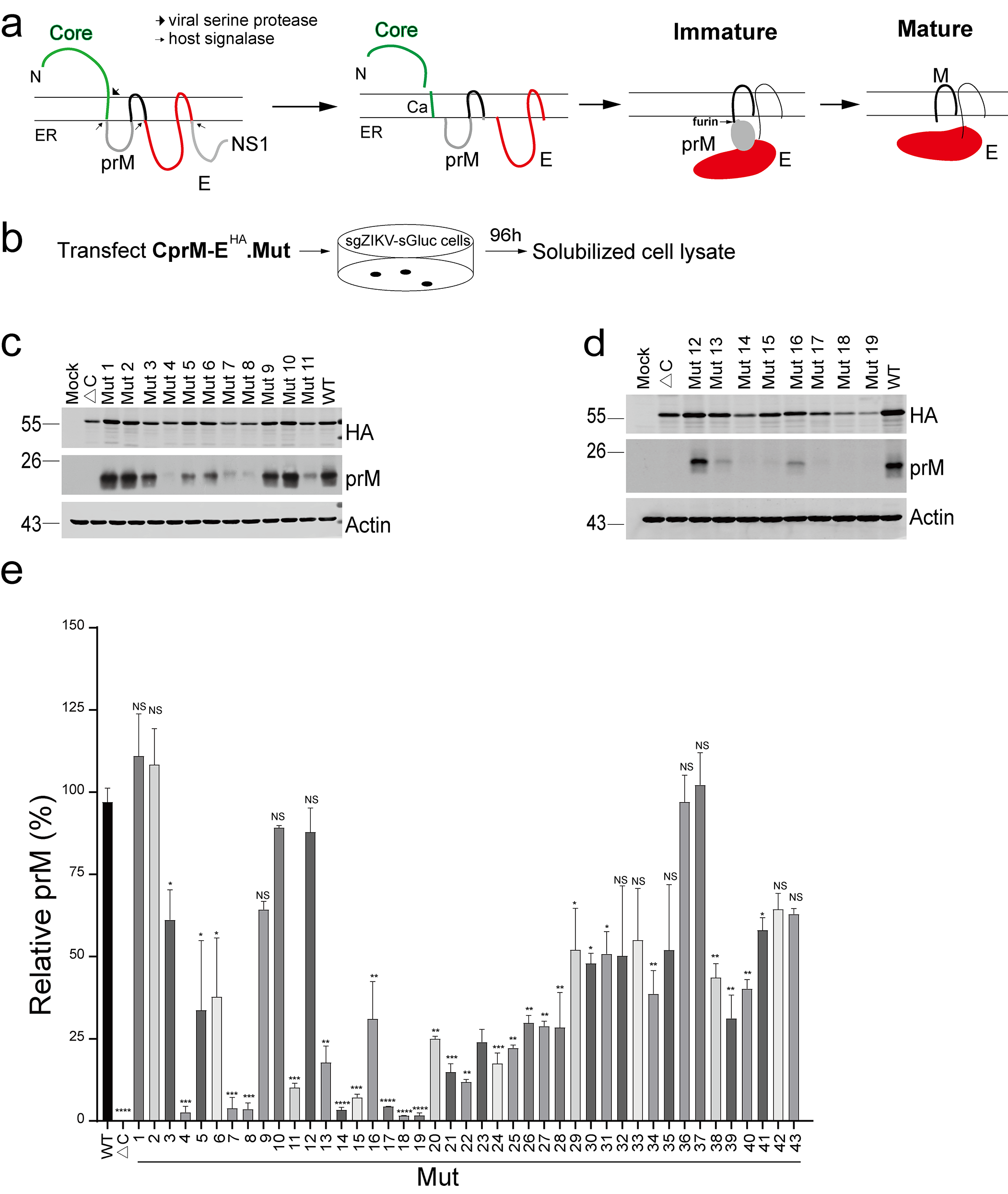


**Supplementary Figure 3. Impact of E mutations on prM expression.** (a) Schematic of Capsid-prM-E processing of flaviviruses. Capsid-prM-E polypeptide is processed by the viral NS3 protease and host signalase. A heterodimer of the processed prM and E assembles into the immature virion that is composed of trimeric prM-E heterodimer. The immature virion matures after processing of prM by furin. Ca, Capsid anchor. ER, endoplasmic reticulum. (b) Schematic of the experimental design for (c-e). Plasmids expressing CprM-E^HA^ (WT), prM-E^HA^ (∆C) and CprM-E^HA^-mutants were transfected into sgZIKV-sGluc-Vero cells. After 96 hours, the cells were solubilized and then analyzed by Western blotting. (c-d) Representative pictures of the Western blotting analysis. The values to the left of the blots are molecular sizes in kilodaltons. (e) The relative abundances of prM (calculated as input prM /Actin) in E-mutant-transfected cells were further normalized to the WT and plotted. Mean values ± SDs are shown (n = 3). Statistical analysis was performed between WT and the mutants as indicated. (ns, not significant, *P < 0.05; **P < 0.01; ***P < 0.001; ****P < 0.0001; two-tailed, unpaired *t*-test).

**Supplementary Figure 4**


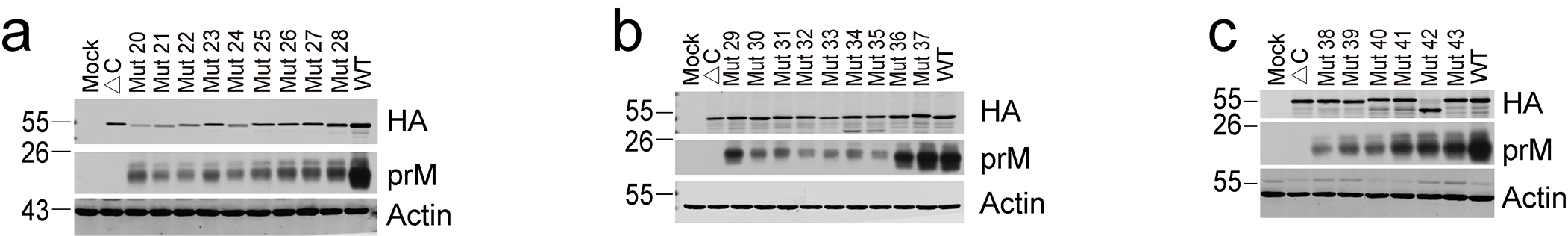


**Supplementary Figure 4. Impact of E mutations on prM expression.** (a-c) Plasmids expressing CprM-E^HA^ (WT), prM-E^HA^ (∆C) and CprM-E^HA^-mutants were transfected into sgZIKV-sGluc-Vero cells. After 96 hours, the cells were solubilized and then analyzed by Western blotting with the indicated antibodies. The values to the left of the blots are molecular sizes in kilodaltons.

**Supplementary Figure 5**


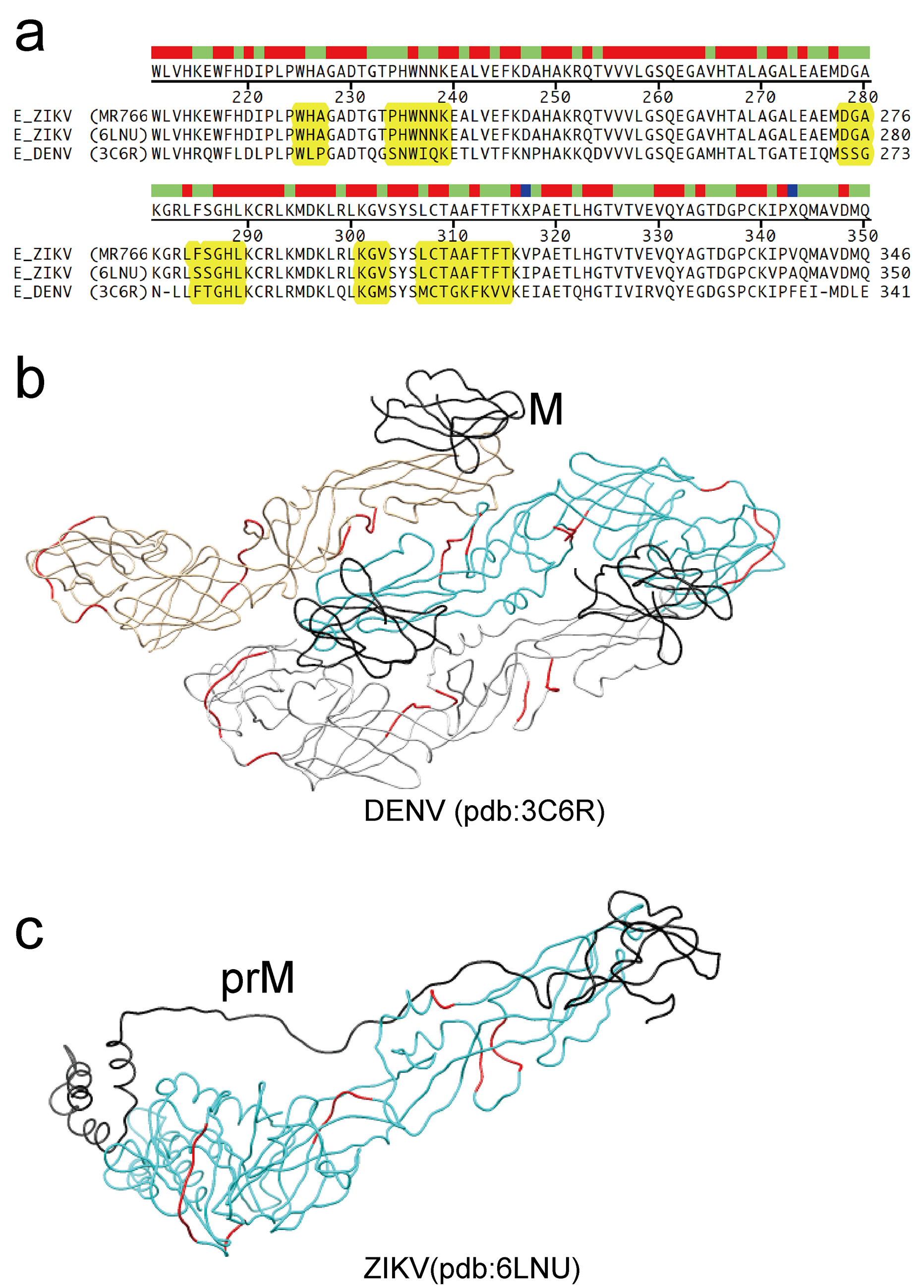


**Supplementary Figure 5. Structural visualization of E mutants that affect prM expression.** (a) Alignment of the residues from ZIKV strains and DENV. Mutated residues corresponding to Mut 4, Mut 7, Mut 8, Mut 11, Mut 13, Mut 14, Mut 15, Mut 16, Mut 17, Mut 18 and Mut 19 are highlighted. (b) The structure of dengue virus (DENV) in low pH (PDB, 3C6R) is shown. The cleaved M (in black) is associated with E. The residues equivalent to the mutated residues are indicated in red. (c) The structure of the ZIKV immature virion (PDB, 6LNU) is shown. The prM are indicated in black. The residues equivalent to the mutated residues are in red.

**Supplementary Figure 6**


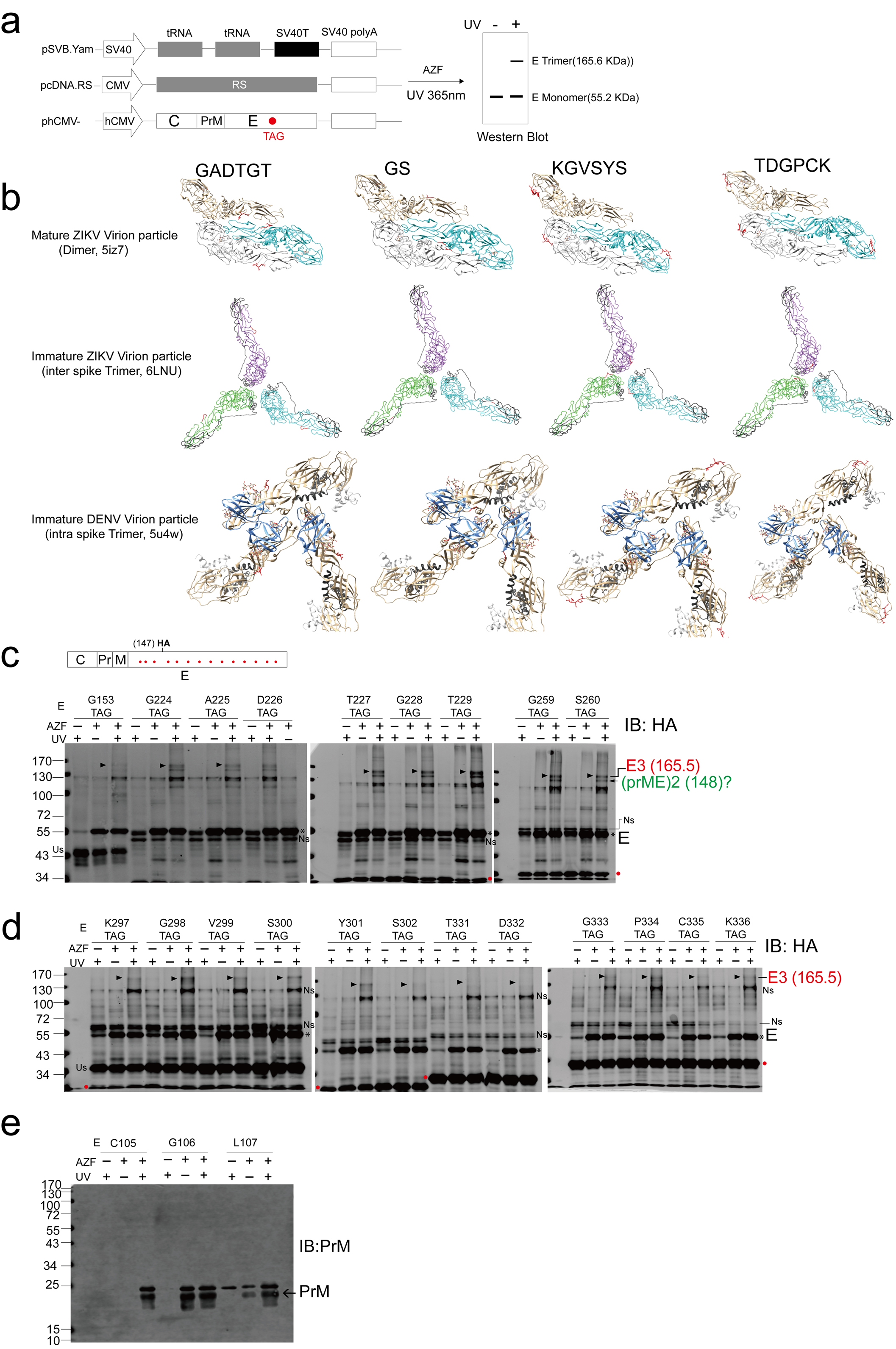


**Supplementary Figure 6. Visualization of E timer by a bioorthogonal system.**  (a) Schematic of the bioorthogonal system used in this study. (b) Localizations of the equivalent residues of the selected residues (in red) in the structures. (c-d) The sgZIKV-sGluc-Vero cell line was cotransfected with the plasmid pSVB.Yam, pcDNA.RS, and plasmids expressing CprM-EHA with the TAG stop codon (red dots) introduced at the indicated residues. After photocrosslinking, the cells were subjected to immunoprecipitation (IP) with anti-HA beads and the immunoprecipitated proteins were analyzed by Western blotting. The arrows indicate the E trimer with the expected molecular weight of 165.5 KD and the putative dimeric prME with the expected molecular weight of 148 KD. The asterisks indicate the E monomers. The red dots indicate the non-AZF-incorporated proteins whose translation is stopped at the introduced TAG codons. Ns, nonspecific bands; Us, unspecified bands. The values to the left of the blots are molecular sizes in kilodaltons. (e) The sgZIKV-sGluc-Vero cell line was cotransfected with the plasmid pSVB.Yam, pcDNA.RS, and plasmids expressing CprM-E^HA^ with the TAG stop codon introduced at the indicated residues. After photocrosslinking, the cells were subjected to immunoprecipitation (IP) with anti-HA beads and the immunoprecipitated proteins were analyzed by Western blotting. The arrows indicate prM monomers, but unfortunately (prME)_2_ cannot be detected by Anti-ZIKV prM (GTX133305; GeneTex), which were used in Western blotting at 1:300 dilution.

**Supplementary Figure 7**


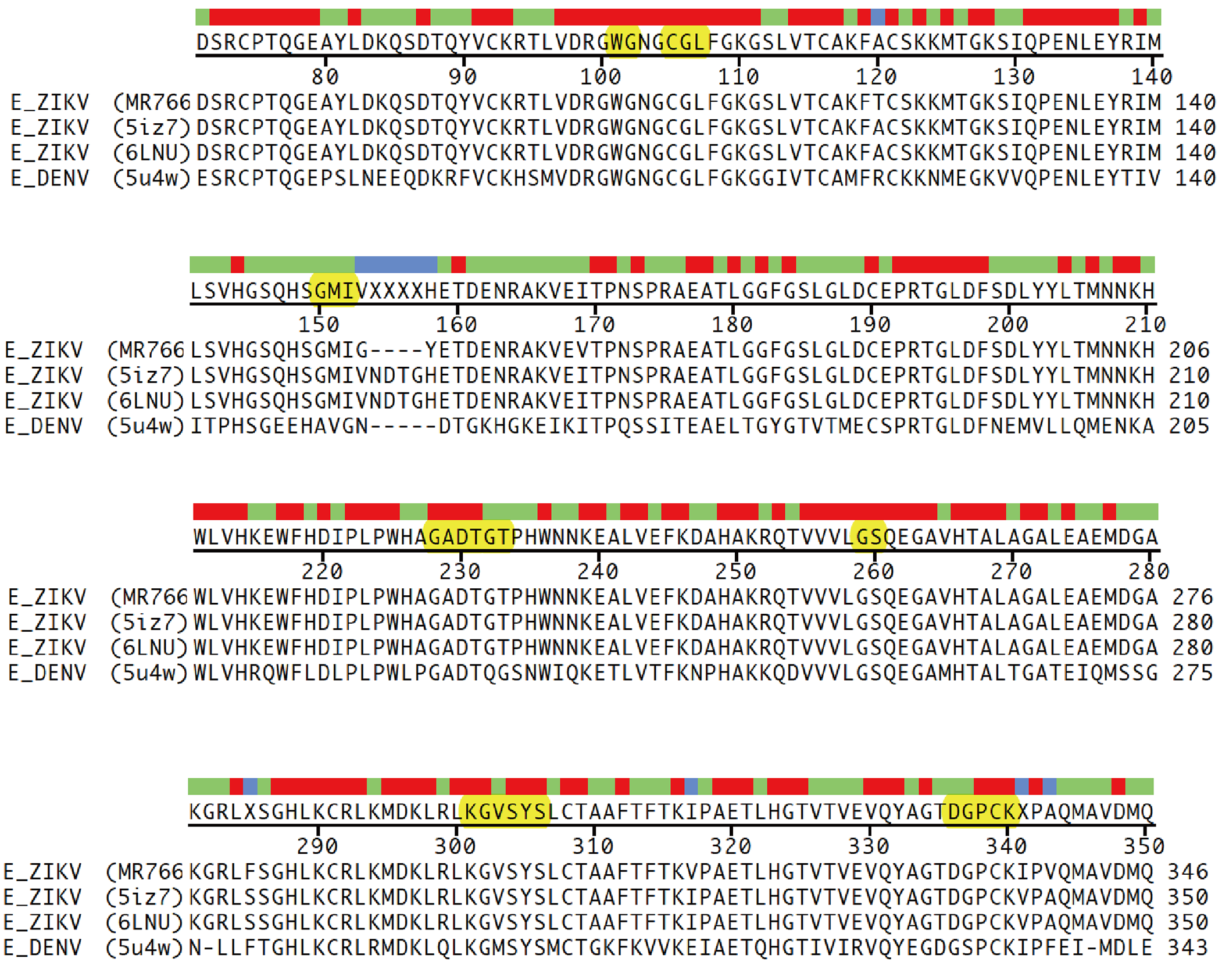


**Supplementary Figure 7. Alignment of ZIKV E and DENV E.** Protein sequences of E from different ZIKV strains and DENV were analyzed by MegAlign (DNAstar). Residues in the interfaces are highlighted.

**Supplementary Figure 8**


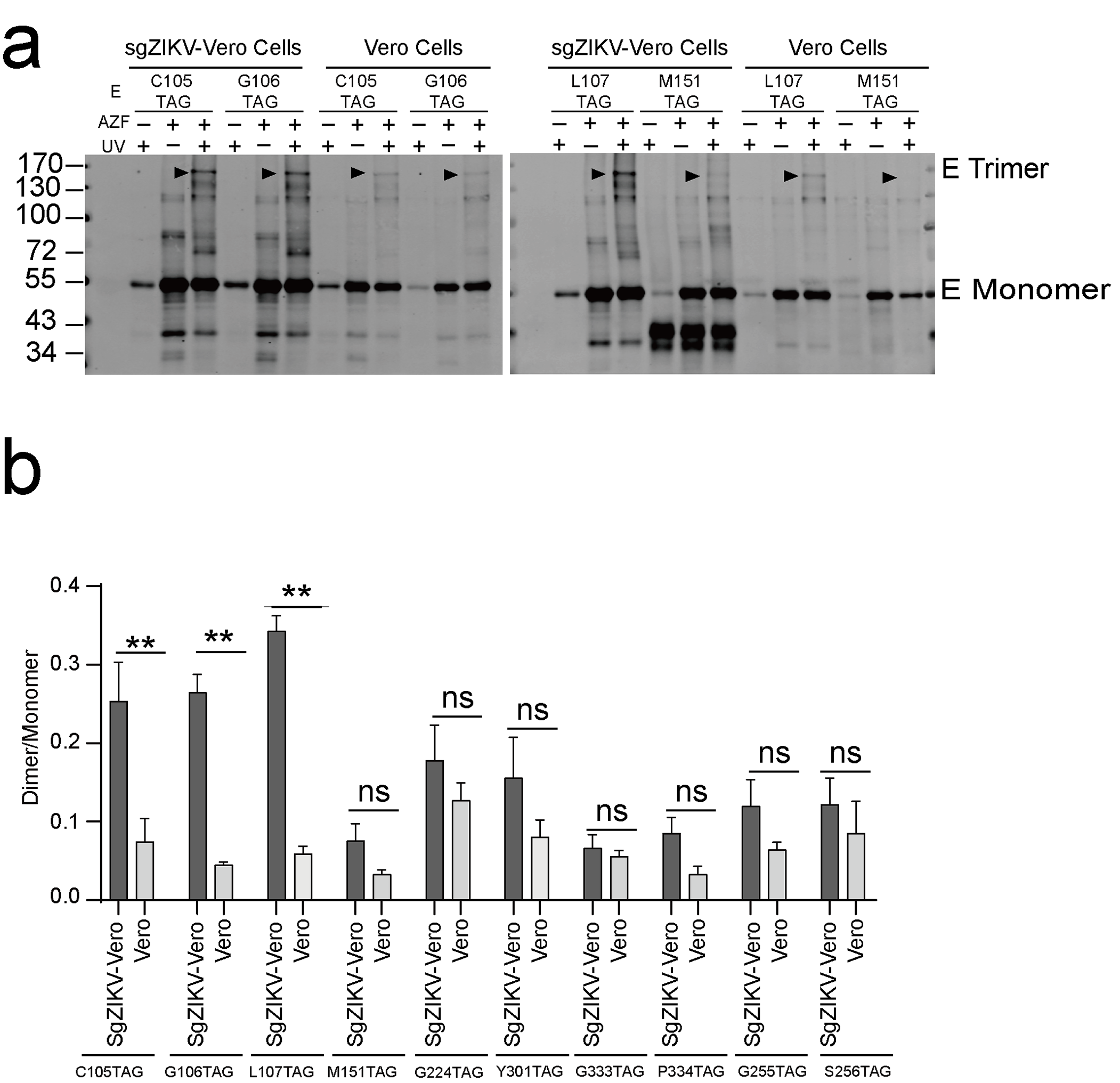


**Supplementary Figure 8. Formation of E trimers in the absence and presence of viral non-structural proteins.** (a) SgZIKV-sGluc-Vero cell line and Vero cells were cotransfected with the plasmid pSVB.Yam, pcDNA.RS, and the plasmids expressing CprM-E^HA^ with the TAG stop codon introduced at the indicated residues. After UV-crosslinking and immunoprecipitation (IP) with anti-HA beads, proteins were analyzed by Western blotting with anti-HA antibody. Arrows indicate the E trimer with the expected molecular weights (165.5 KD). (b) The abundances of the E trimer in A were quantified, and the intensity of the E trimer (E trimer) / (E monomer) after UV exposure was calculated. Data combined from 2 independent experiments are shown (mean ± SEM, n=4). Statistical analysis was performed between the sgZIKV-Vero cells and Vero cells as indicated. (ns, not significant, **P < 0.01; two-tailed, unpaired t-test).

**Supplementary Figure 9**


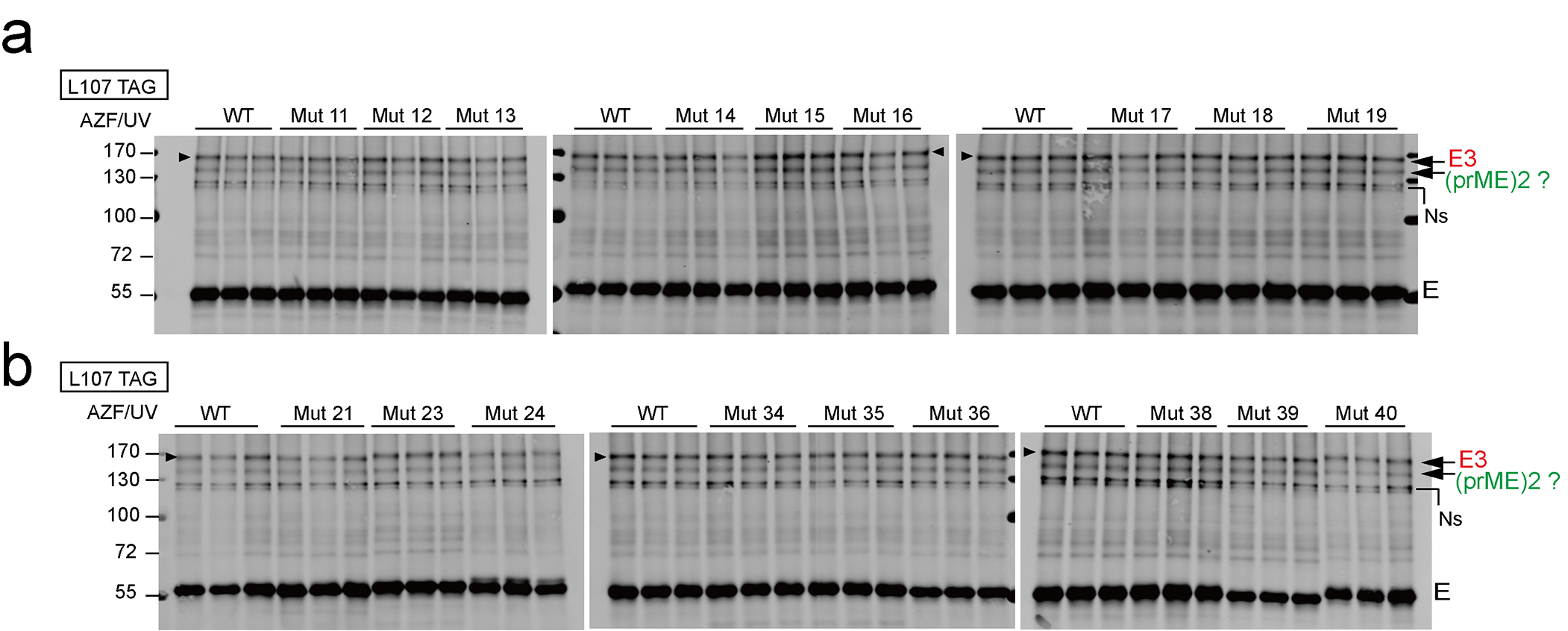


**Supplementary Figure 9. Impact of E mutations on E trimerization.** (a-b) SgZIKV-sGluc-Vero cells were cotransfected with the plasmid pSVB.Yam, pcDNA.RS and the E mutant plasmids with the TAG stop codon introduced in L107. After UV-crosslinking, the cell lysates were subjected to immunoprecipitation (IP) with anti-HA beads. Proteins were analyzed by Western blotting with anti-HA antibody. Samples from triplicate wells were shown. The E trimers (E3) and the putative dimeric prME are indicated. Ns, nonspecific bands. The values to the left of the blots are molecular sizes in kilodaltons.

**Supplementary Figure 10**


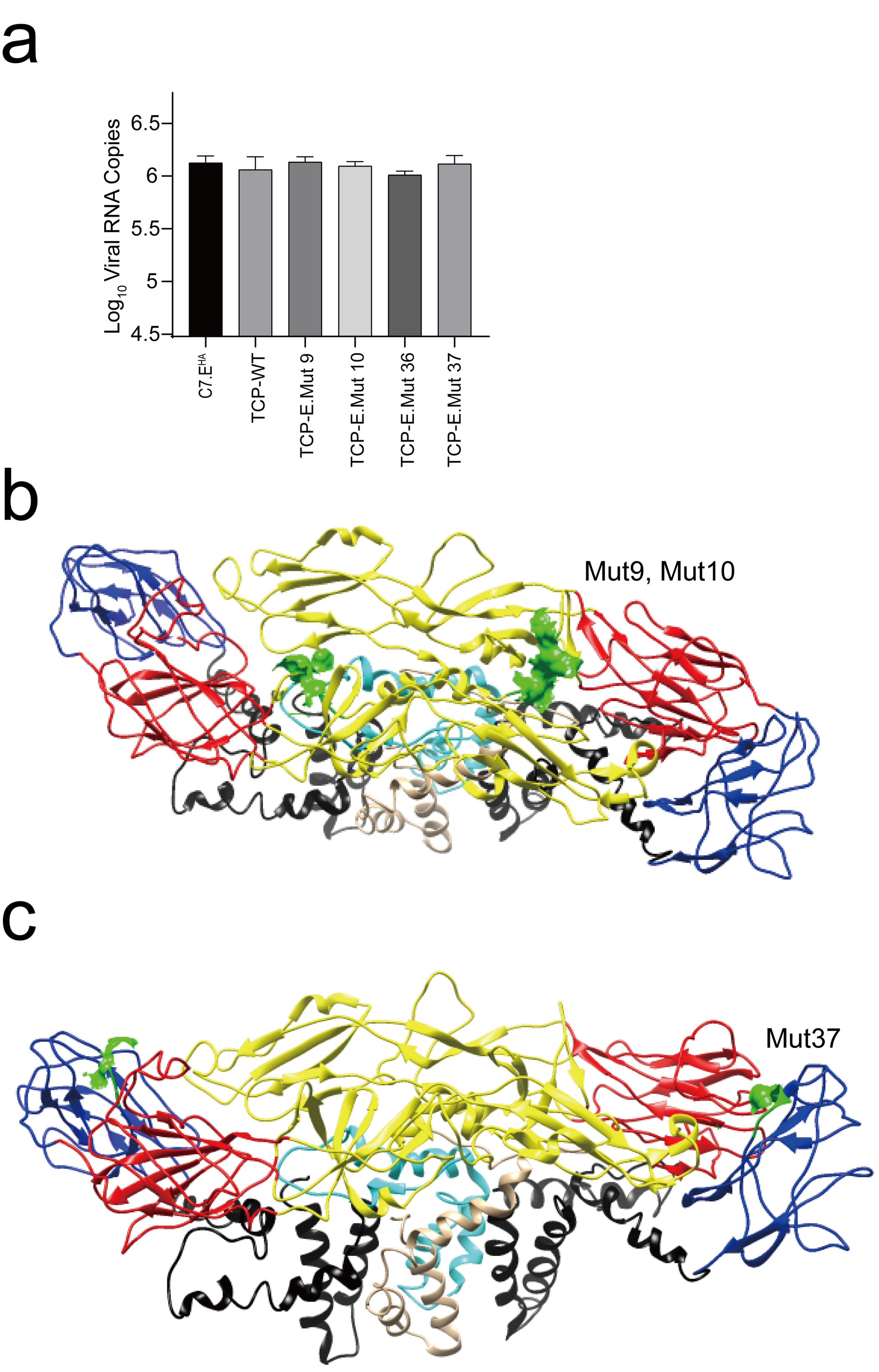


**Supplementary Figure 10. Locations of Mut9, Mut10 and Mut37 on the E structure.** (a) RNA analysis concentrated ZIKV_TCPs_. (b and c) The equivalent residues to Mut9 and Mut10 (in green) (b) and Mut37 (in green) (c) in the ZIKV E structure (PDB, 5i7z) are shown.

**Supplementary Figure 11**


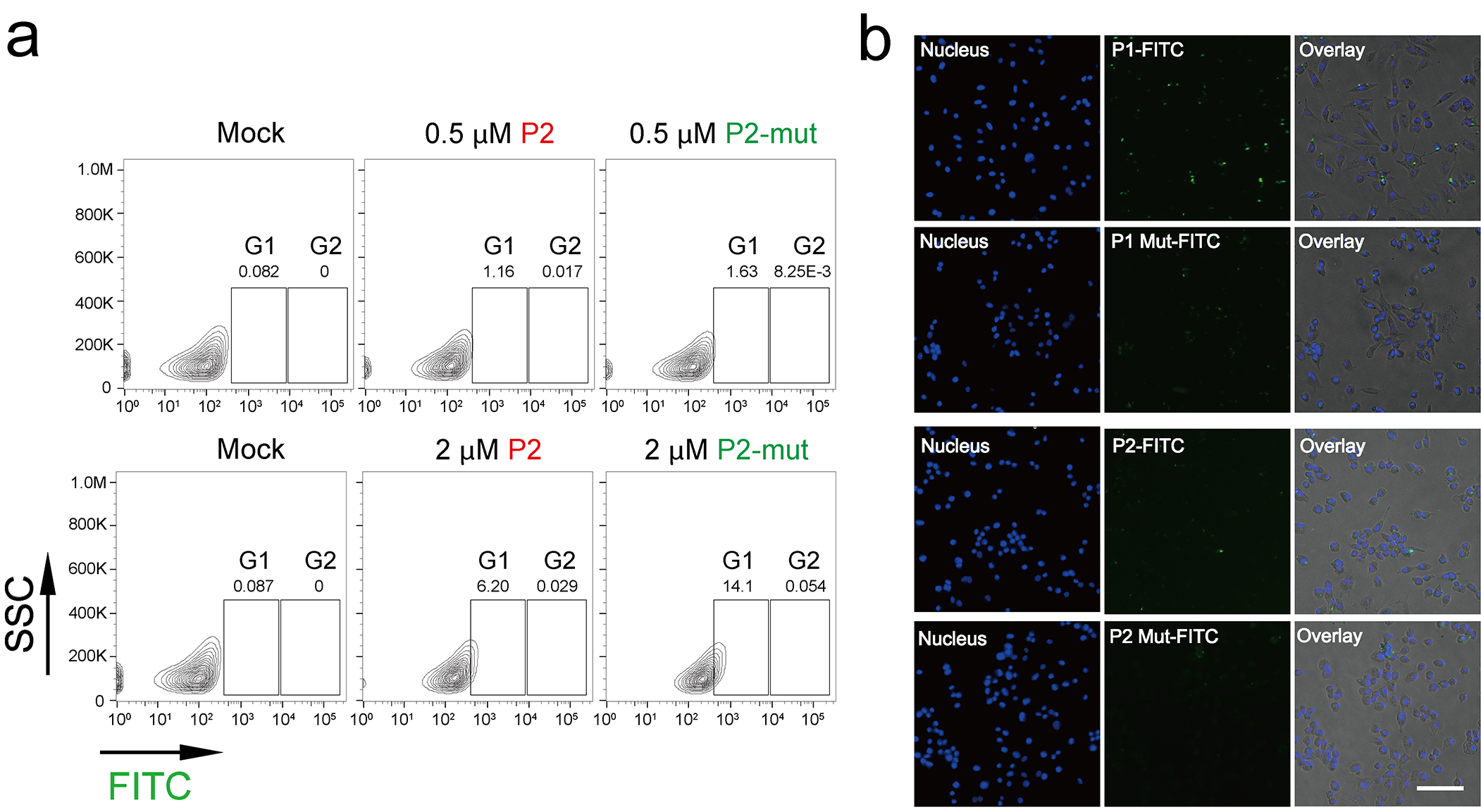


**Supplementary Figure 11. Binding of ZIKV E-derived Peptides to Vero cells.** (a) Vero cells were incubated with PBS (Mock) and various concentrations of FITC-conjugated P2 and P2-Mut for 2 hours at 4 °C. Then the cells were washed, fixed and analyzed by flow cytometry. (b) Vero cells were incubated with PBS (Mock), 10 μM FITC-conjugated peptides P1 and P1-Mut or 50 μM FITC-conjugated peptides P2 and P2-Mut for 2 hours at 4 °C and then fixed. After staining the nucleus with Hoechst 33342, the cells were observed by fluorescence microscopy. Scar bar, 100 μM.

**Supplementary Figure 12**


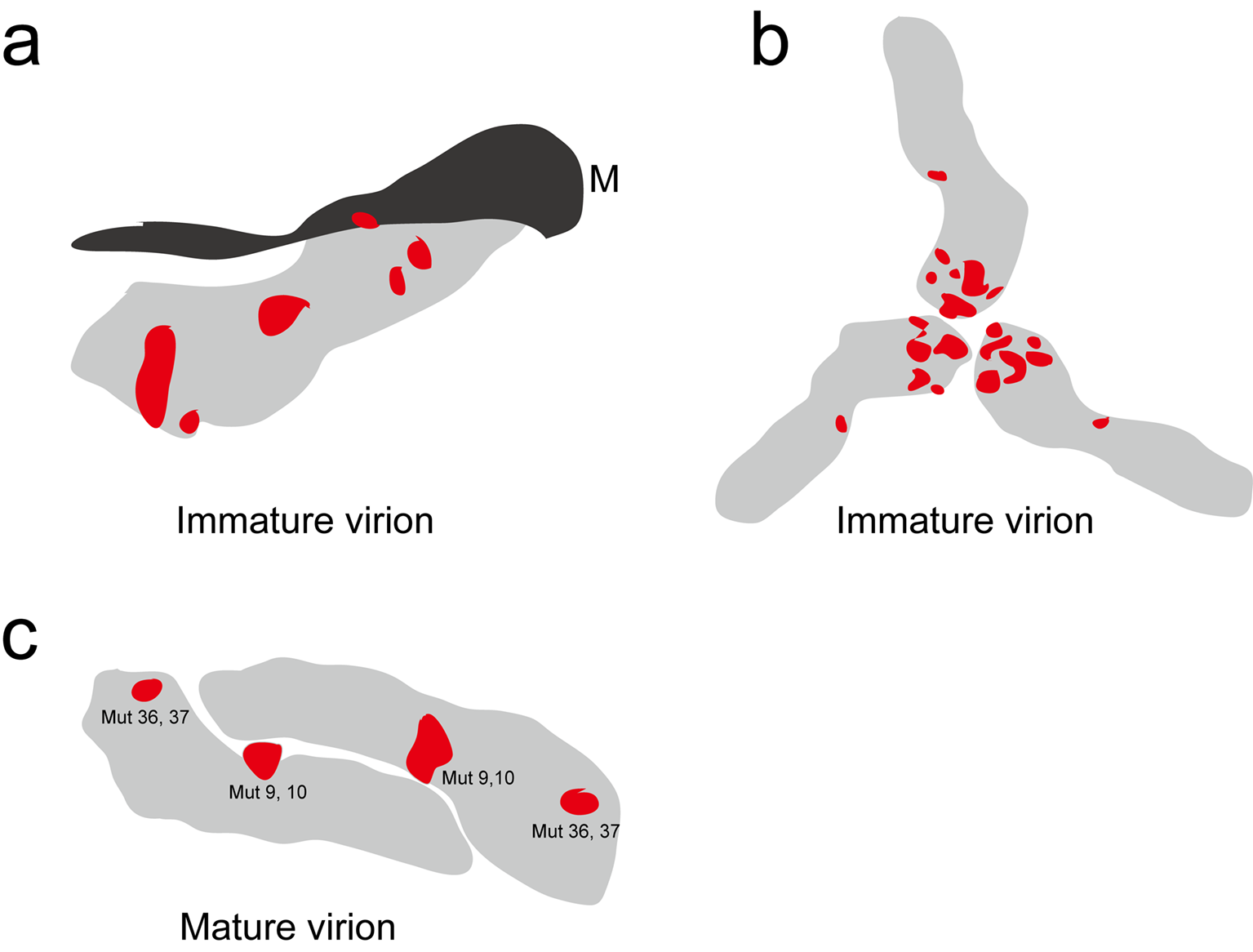


**Supplementary Figure 12.** (a) Schematic of the mutants that reduce prM expression on the ZIKV immature virion. The locations of these mutants (Mut 4, Mut 7, Mut 8, Mut 11, Mut 13, Mut 14, Mut 15, Mut 16, Mut 17, Mut 18 and Mut 19) are indicated in red. The prM is in black. (b) Schematic of the mutants that reduce E trimerization on the ZIKV immature virion. The locations of these mutants (Mut 9, Mut10, Mut14, Mut 20, Mut 22, Mut 27, Mut 37, Mut 40 and Mut 42) are indicated in red. (c) Schematic of the mutants that impair viral attachment on the ZIKV immature virion. The locations of these mutants (Mut 9, Mut 10, Mut 36, Mut 37) are indicated in red.
