# Supplementary material for: Identification and characterization of key residues in Zika virus envelope protein for virus assembly and entry
Source: Emerg Microbes Infect. 2022 Jun 10;11(1):1604–20. doi: 10.1080/22221751.2022.2082888 (PMC9196690; doi:10.1080/22221751.2022.2082888)
Supplement: Supplemental Material [file TEMI_A_2082888_SM5429.zip › Supplementary table 2.docx]

**Supplementary table 2. E Mutants and the mutated residues**

| Domain | Mutant | Residues |
| --- | --- | --- |
| Domain I | Mut 1 | *M151A/I152A/G153A* |
|  | Mut 2 | *Y154A/E155A/T156A* |
|  | Mut 3 | *D157A/E158A/N159A* |
|  | Mut 15 | *K297A/G298A/V299A* |
|  | Mut 16 | *S300A/Y301A/S302A* |
| Domain II | Mut 4 | *W221A/H222A/A223* |
|  | Mut 5 | *G224A/A225/D226A* |
|  | Mut 6 | *T227A/G228A/T229A* |
|  | Mut 7 | *P230A/H231A/W232A* |
|  | Mut 8 | *N233A/N234A/K235A* |
|  | Mut 9 | *A268/L269A/E270A* |
|  | Mut 10 | *A271/E272A/M273A* |
|  | Mut 11 | *D274A/G275A/A276* |
|  | Mut 12 | *K277A/G278A/R279A* |
|  | Mut 13 | *L280A/F281A/S282A* |
|  | Mut 14 | *G283A/H284A/L285A* |
| Domain III | Mut 17 | *L303A/C304A/T305A* |
|  | Mut 18 | *A306/A307/F308A* |
|  | Mut 19 | *T309A/F310A/T311A* |
|  | Mut 20 | *K312A/V313A/P314A* |
|  | Mut 21 | *A315/E316A/T317A* |
|  | Mut 22 | *H319A/G320A/T321A* |
|  | Mut 23 | *V322A/T323A/V324A* |
|  | Mut 24 | *E325A/V326A/Q327A* |
|  | Mut 25 | *Y328A/A329/G330A* |
|  | Mut 26 | *T331A/D332A/G333A* |
|  | Mut 27 | *P334A/C335A/K336A* |
|  | Mut 28 | *I337A/P338A/V339A* |
|  | Mut 29 | *M345A/Q346A/T347A* |
|  | Mut 30 | *L348A/T349A/P350A* |
|  | Mut 31 | *V351A/G352A/R353A* |
|  | Mut 32 | *L354A/I355A/T356A* |
|  | Mut 33 | *A357/N358A/P359A* |
|  | Mut 34 | *V360A/I361A/T362A* |
|  | Mut 35 | *E363A/S364A/T365A* |
|  | Mut 36 | *E366A/N367A/S368A* |
|  | Mut 37 | *K369A/M370A/M371A* |
|  | Mut 38 | *L372A/E373A/L374A* |
|  | Mut 39 | *D375A/P376A/P377A* |
|  | Mut 40 | *Y382A/I383A/V384A* |
|  | Mut 41 | *I385A/G386A/V387A* |
|  | Mut 42 | *G388A/D389A/K390A* |
|  | Mut 43 | *K391A/I392A/T393A* |
